# Regime shifts in marine communities: a complex systems perspective on food web dynamics

**DOI:** 10.1098/rspb.2015.2569

**Published:** 2016-02-24

**Authors:** Johanna Yletyinen, Örjan Bodin, Benjamin Weigel, Marie C. Nordström, Erik Bonsdorff, Thorsten Blenckner

**Affiliations:** 1 Stockholm Resilience Centre, Stockholm University, Stockholm, Sweden; 2 Environmental and Marine Biology, Åbo Akademi University, Turku, Finland

**Keywords:** regime shift, complex adaptive systems, exponential random graph model, Baltic Sea, food web, motifs

## Abstract

Species composition and habitats are changing at unprecedented rates in the world's oceans, potentially causing entire food webs to shift to structurally and functionally different regimes. Despite the severity of these regime shifts, elucidating the precise nature of their underlying processes has remained difficult. We address this challenge with a new analytic approach to detect and assess the relative strength of different driving processes in food webs. Our study draws on complexity theory, and integrates the network-centric exponential random graph modelling (ERGM) framework developed within the social sciences with community ecology. In contrast to previous research, this approach makes clear assumptions of direction of causality and accommodates a dynamic perspective on the emergence of food webs. We apply our approach to analysing food webs of the Baltic Sea before and after a previously reported regime shift. Our results show that the dominant food web processes have remained largely the same, although we detect changes in their magnitudes. The results indicate that the reported regime shift may not be a system-wide shift, but instead involve a limited number of species. Our study emphasizes the importance of community-wide analysis on marine regime shifts and introduces a novel approach to examine food webs.

## 1. Introduction

Ever-increasing anthropogenic activities such as overfishing, invasive species transport, pollution and climate change can drive marine systems to suddenly shift to new regimes (states) with often devastating effects for human communities relying on marine resources for their persistence [1]. Despite the severe nature of regime shifts, here described as a persistent change in the structure and dynamics of the whole system [1], knowledge on how to explicitly define and detect them, their underlying causal mechanisms, and the ability to predict potentially forthcoming shifts is largely in its infancy. In particular, empirical research on marine regime shifts has proved to be difficult [2]. This gap can at least partly be ascribed to the analytically and empirically challenging task of trying to reveal underlying patterns and processes in large and complex datasets such as food webs (cf. [3]). Therefore, in practice, any large, abrupt and persistent change in any of the key state variables describing a system is often conceived as a regime shift [4]. Although this empirically oriented research has provided many important insights into if and how regime shifts can emerge, it is still largely an open question to what extent these reported regime shifts pervade the whole system, or if they mainly affect a limited part of the system. To arrive at a more fundamental understanding of the underlying causes and mechanisms behind system-wide regime shifts, we need to embrace a whole-system approach in researching regime shifts (cf. [3]). The aim of this work is to show how complexity theory, in combination with a recently developed network modelling framework, can be used to disentangle the underlying interactions of species in system-wide food webs before and after a reported regime shift in the Baltic Sea.

Complexity theory provides a conceptual theoretical framework, upon which system-wide changes can be defined and understood [5,6]. Complexity theory is a wide and to some extent scattered field, but here we will mainly draw on the perspective of complex adaptive systems (CAS) [7]. At the core of CAS lies the assumption that complex systemic behaviours emerge from interactions between often numerous interacting components (agents) [8,9]. Changes in these micro-level processes can, at some critical point, build up and interact in ways that cause a major shift at the system level [5]. In addition, it is assumed that most complex systems are controlled by a fairly limited number of such micro-level processes [8]. Thus, a regime shift can be understood as when underlying agents interact, defined through a *limited* set of micro-level processes, in such ways as to push the system beyond a critical threshold where these changes will escalate and finally drive the whole system into a new regime. We however acknowledge that other conceptualizations of regime shifts do exist within the broader complexity literature. Nonetheless, complexity science in large part suggests that systems can be understood as networks of interacting components, and the complex pattern in which these components interact with each other can inform on large-scale systems behaviour (a cornerstone assumption in the new science of complex networks [9]).

**Figure 1.**
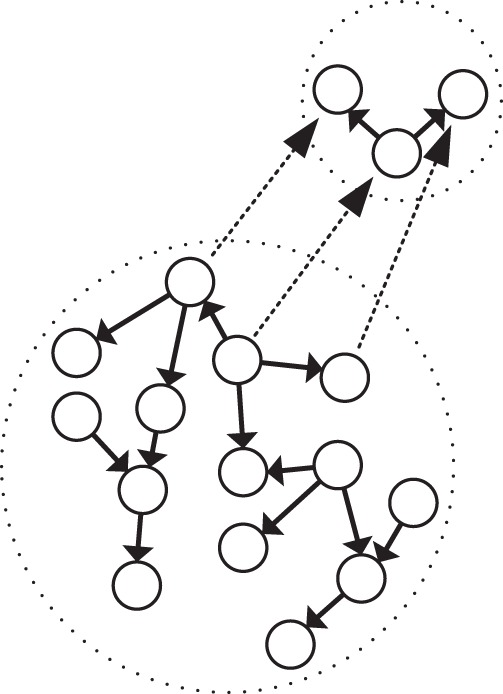
A motif as a network substructure.

Ecosystems constitute prototypical examples of CAS [7], and are often described as complex networks composed of species (nodes), and trophic interactions (links) that link the species together in an ecological community (i.e. food web). Diverse sets of empirical food webs share similar micro-level substructures that occur more (or less) often than expected by chance [10,11]. These frequently occurring network substructures are identified as network motifs (figure 1) [10], and can be associated with the various micro-level processes that, as postulated in CAS theory, together define behaviour at the systems level [7,10]. In essence, a motif is seen as the structural representation of a process that is defined through the ways nodes interact (table 1). For example, if there was a process, which makes nodes respond to an incoming link with an outgoing link, one would expect to find prevalence for motifs consisting of two nodes connected through a bi-directed link. The system-level implications of the prevalence (or lack) of certain motifs have been demonstrated in simulation studies that, for example, show that the stability of food webs is seemingly dependent on the food webs' compositions of these substructures [27,28]. Such local to global scale approaches clearly have implications for understanding and predicting regime shifts, and represent an effort to bring insights from complexity theory to community ecology.

**Table 1.** Species interaction processes, the corresponding motifs and ERGM configurations. The names of the ERGM configurations as they are called in MPNET are inside parentheses. Note that the ERGM configurations used here are configurations that consist of a series of simpler configurations, the ‘alternate’ version of a configuration that still captures the same type of underlying process as in the more bare-bone configuration, see further [12]. The reason for using the alternate configurations lies in the degree heterogeneity: in empirical networks triangles tend to clump together instead of being evenly distributed throughout the network, thus alternating configurations improve the ability of ERGM to reproduce empirical network structure improves significantly [12,13].

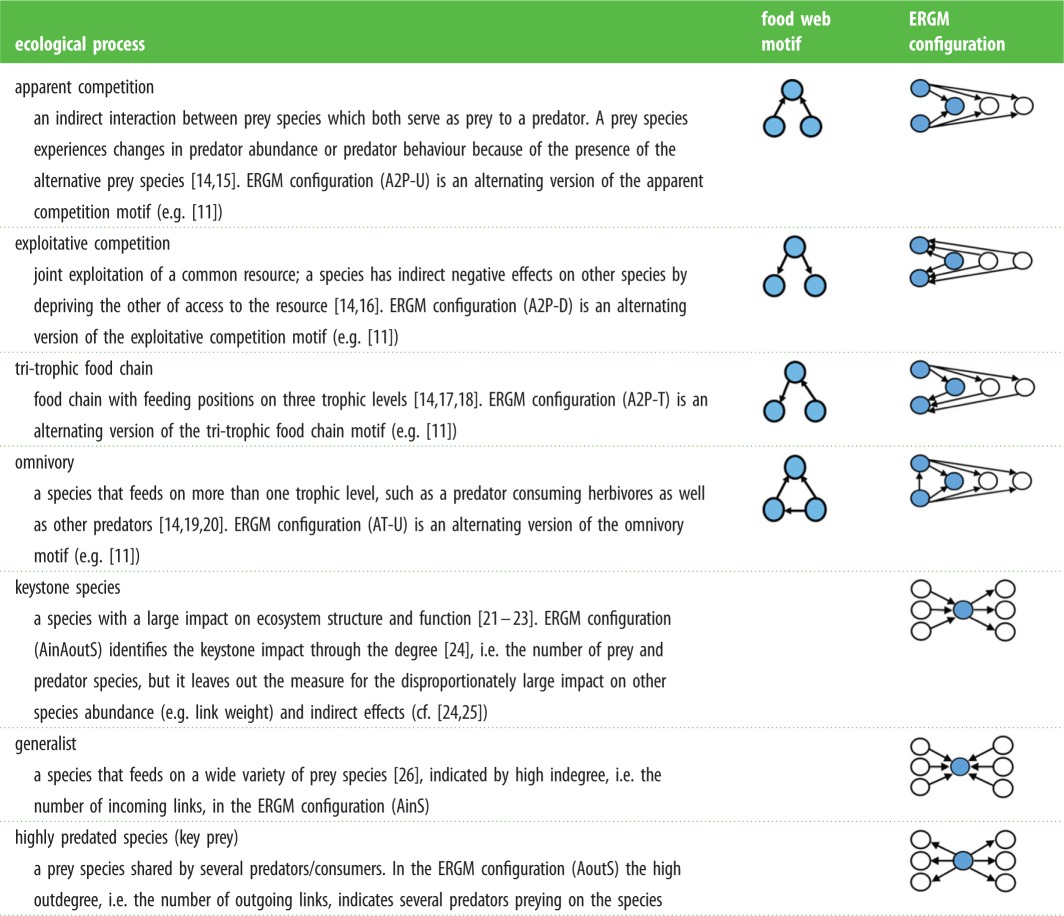

In spite of the important insights previous motif-based studies of food webs have provided [29], these earlier approaches have a limitation of being merely descriptive when statistically analysing the structures of the observed food webs. Therefore, the ability to rigorously differentiate between the prevalence of certain motifs as being the cause or the effect of any real underlying processes is inherently limited. Without such knowledge, the ability to develop a more theoretically informed process-based understanding of regime shifts, why they occur, and how they can be predicted, is severely hampered.

Our overall aim is to address this gap by re-interpreting patterns of motif occurrences in species interactions in food webs through applying a recent network modelling framework developed in the social sciences (exponential random graph models (ERGM^1^), e.g. [12]). ERGM is here used to discern the minimal (core) set of underlying processes of species interactions in an ecological community and how these processes give rise to complex food web structures. Thus ERGM, in agreement with the theoretical foundations of CAS theory, provides an analytically and empirically traceable means to differentiate between causes and effects when linking the observed structure of a system (described as a network of interacting components) with a parsimonious set of underlying micro-scale processes.

ERGM, like previous motif-based studies, builds on the conceptualization of the network being built up by different micro-scale network substructures (motifs). In ERGM terminology, these network substructures are usually called *configurations* instead of motifs*.* We will use that term unless we explicitly refer to earlier motif-based approaches. Motif-based approaches calculate the frequency of different configurations in the empirical network and compare these frequencies with the same configurations' frequencies derived from a large set of random networks (thus representing the null model). ERGM differs from this descriptive approach in that it encourages and supports identifying the *minimal* but *sufficient* (core) set of micro-level configurations that explains the whole structure of the network. Hence, ERGM indirectly takes into account ‘dynamic effects' in that the prevalence of specific configurations in shaping the structure of the network can give rise to over/underrepresentation of other configurations. ERGMs are in a sense conceptually similar to multivariate regression models, where the explanatory variables are represented by a set of configurations, and the dependent variable is the network itself. As when using multiple predictors in a regression, multiple configurations in ERGM can be considered together to examine which are most important in explaining the structure of the entire network. To emphasize this even further, when fitting an ERGM to empirical data, the aim is to make empirical inferences about those configurations required to explain the structure of the network: the frequency of other configurations can be interpreted as a secondary phenomenon/effect. This ability to differentiate between configurations is an important feature that distinguishes ERGM from earlier approaches.

In other words, ERGM encourages a process-oriented approach, which is more congruent with CAS theory in that it supports the identification of these specific configurations that represent the underlying *processes* that together give rise to the observed system-level network structure. In addition, ERGM facilitates off-the-shelf analyses of configurations that have not previously been conceptualized as motifs [12]. Finally, ERGM also makes use of a frequency analysis of a large set of configurations, but this only constitutes the final step in the ERGM modelling process where the estimated network model is validated (‘goodness of fit’; GoF). (See the electronic supplementary material for further descriptions of ERGM.)

We use the Baltic Sea as a case study to demonstrate the applicability of ERGM in empirical research on regime shifts in marine systems. The Baltic Sea is a heavily exploited sea that experienced rapid, persistent and large changes in some key state variables (e.g. fishing pressure, dominant species, temperature) in the late 1980s, and thus is presumed to have undergone a regime shift [30–32]. We show how models based on ERGM constitute plausible theoretical hypotheses for which micro-level species interaction processes have led to the empirically observed food webs, before and after the reported regime shift [12]. Hence, comparisons of food web ERGMs at these different points in time allow us to associate potential changes in the way species interact (processes) with the reported regime shift. To our knowledge, this is the first time that ERGM has been applied to food webs (see however [33] for a conceptually similar modelling approach building and a continuous-time Markov chain model of network evolution).

## 2. Material and methods

We constructed four food webs using sampling data (when available), literature and expert opinions. Two food webs were constructed for the offshore area in the central Baltic Sea, representing the decades before and after the documented regime shift, i.e. the 1980s versus the 2000s, and two food webs were constructed for the same time periods for the coastal region of the Åland Islands (see the electronic supplementary material for food web construction as well as electronic supplementary material, table S2 and figure S1*a–d*) in the entrance to the Northern Baltic Sea. The food webs are directed networks with circa 30 nodes and (un-weighted and directed) links based on predation–prey interactions in which the link (arrow) is the direction of energy transfer, namely from prey to predator. Cannibalism is neglected, and thus intra-specific size-structured interactions are not included. The selection of species was based on their relative biomasses within their functional group. In this way, the constructed food webs reflect changes in species biomass.

The essence of ERGM is the underlying assumption that by analysing the patterns of network links, the processes that gave rise to the structure of the network can be revealed [34]. The overall analytical approach is divided into two steps. The first step involves searching for a well-fitting statistical model for an empirical network by selecting and estimating the driving/inhibiting/neutral effect and magnitude of a limited set of configurations. The second step uses simulation techniques to evaluate the robustness of the model in step one, and how well the resulting model is able to reproduce the structural characteristics of the empirical data (GoF) [12].

ERGM is a stochastic model in which links among network nodes are random variables. The probability of a given network *G* is given by a sum of network statistics (*zs*) weighted by parameters (*θ*s) inside an exponential and *c* is a normalizing constant [12]
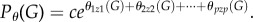


Each network statistics corresponds to a specific network configuration. Similar to multivariate regression analysis, ERGM assesses the effect of each selected core configuration in explaining the observed network structure through parameter estimates. A parameter estimate signifies the magnitude, driving/inhibiting character and the significance of a configuration, given the presence of other selected configurations [12]. For instance, the presence of a (predator) species with large numbers of prey species in a food web causes the ERGM to assign a positive (and likely significant) parameter value for the specific configuration capturing a star-like process with the predator species in the middle (generalist configuration).

In ERGM, as in multivariate regression analysis, one needs to distinguish between the significance of the individual configuration and the appropriateness of a specific model specification taking the ensemble of configuration into account. A parameter estimate for a given configuration depends on which other configurations are included in the model. This is a fundamental difference of ERGM compared with a descriptive frequency analysis where the count of any given motif is independent of the count of other motifs. Further, even though ERGMs are conceptually similar to multivariate regression models, this similarity is merely conceptual. Multivariate regression models assume data independency, whereas network models like ERGM assume the network is formed through processes of interaction. Also, ERGMs are in general capable of picking apart highly correlated effects deriving from configuration entanglements (i.e. when one configuration is structurally entangled in another configuration, like an open triangle being part of a closed triangle).

The GoF procedure is performed to assess how well the model manages to capture the observed frequencies of various configurations of the empirical network that both were, and were not, explicitly modelled [12]. If the model has succeeded in replicating the network structure, the structural statistics of the fitted model are consistent with the corresponding statistics of the empirical network. A well-fitting model therefore represents a plausible theoretical hypothesis for the (core set of) processes that have led to the observed network [34]. Our GoF assessment was based on comparing 32 different configurations in the empirical network with a large assemble of networks generated by the fitted ERGM (that never explicitly modelled more than six different configurations). If the generated set of networks manage to capture the statistical distribution of all these 32 configurations, the fit was assumed to be good. The way we applied ERGM is further detailed in the electronic supplementary material. All ERGM analyses were performed with MPNET software (http://sna.unimelb.edu.au/PNet, see the electronic supplementary material for further details).

Finally, we studied the potential regime shift changes. Parameter estimations from each Baltic Sea ERGM model were compared and the difference from one regime to another was calculated as the percentage difference in parameter values from the 1980s to 2000 (electronic supplementary material, table S7). Offshore and coastal food webs are compared separately, following the cross-system consideration guidelines [12].

## 3. Results

Earlier research has identified tri-trophic food chains, omnivory, apparent competition and exploitative competition (table 1) as the four most common three-node motifs across a range of food webs [11,29]. Hence, we started by testing whether the ERGM configurations equivalent to the four most common food web motifs would also explain the entire food web structure, following the logic of the ERGM approach. We found that the best fitting ERGMs include two to three additional configurations (albeit not all configurations were individually significant). This suggests that the set of underlying processes that give rise to the observed food webs is larger than the set of the four most common motifs.

**Figure 2.**
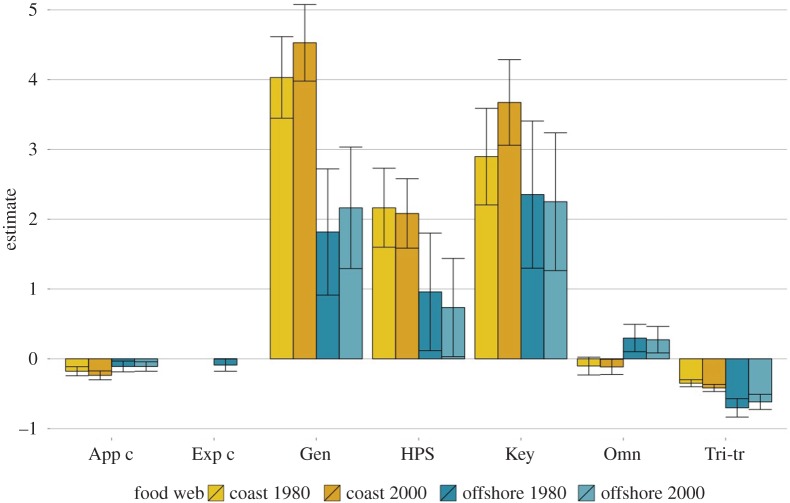
The driving configurations of the Baltic Sea ERGMs, presented here as parameter estimates. ‘App c’: apparative competition, ‘Exp c’: exploitative competition, ‘Gen’: generalist, ‘HPS': highly predated species, ‘Key’: keystone species, ‘Omn’: omnivory, ‘Tri-tr’: tri-trophic food chain. The error bar indicates standard error for parameter estimate, linked to the significance of the configuration (cf. [12]).

The configurations explaining the Baltic Sea food web structures are tri-trophic food chain [14,17,18], apparent competition [15,35], keystone species [21–23], highly predated species, generalists [26], omnivory [14,19,20] and exploitative competition [14,16] (illustrated in table 1 and figure 2). This set of configurations uncovered by the ERGM replicates the food web structure adequately across all four Baltic Sea food webs despite changes in species composition involving nine species in the coastal region, and addition of species; three in the offshore and one in coastal 2000s food webs. In other words, our analysis suggests that these different configurations (processes), to a varying degree (as captured by parameter estimate and level of significance), are themselves driving the formation of the entire food web structures for both the coastal and open water areas, before and after the observed regime shift (table 2).

**Table 2.** Parameter estimates for the Baltic Sea ERGM configurations. A parameter estimate indicates the magnitude and significance of the configuration, giving the presence of other selected configurations. A positive value means driving, and a negative value inhibiting character of the configuration. ‘App c’: apparent competition, ‘Exp c’: exploitative competition, ‘Gen’: generalist, ‘HPS': highly predated species, ‘Key’: keystone species, ‘Omn’: omnivory, ‘Tri-tr’: tri-trophic food chain. Arc is a baseline propensity for the occurrence of the ties, and is not considered a driving configuration [12]. It represents a single link, and is included in the models to control for varying densities (albeit not a direct measure for network density).

	offshore 1980s	offshore 2000s	coast 1980s	coast 2000s
arc	−5.1398	−5.7522^a^	−11.3957^a^	−12.0156^a^
generalist	1.8165^a^	2.1638^a^	4.0303^a^	4.5274^a^
highly predated species	0.959	0.7339	2.164^a^	2.0822^a^
keystone species	2.3523^a^	2.2518^a^	2.8967^a^	3.6722^a^
omnivory	0.2981	0.2741	−0.1033	−0.1157
tri-trophic food chain	−0.702^a^	−0.617^a^	−0.3501^a^	−0.4182^a^
apparent competition	−0.1096	−0.1099	−0.1779^a^	−0.2373^a^
exploitative competition	−0.0888			

^a^Significant configurations.

Table 2 shows that three and five configurations in offshore and coastal food webs, respectively, have individual significant effects on the food web structure. In the offshore food webs, tri-trophic food chains have a significant negative effect and generalists and keystone species have significant positive effects on the food web structure. Thus, this ecological community is characterized by a tendency for avoiding the formation of tri-trophic chains, but rather with a tendency for generalist and keystone species to emerge. In the coastal food webs generalists, keystone species and highly predated species have significant positive effects. Tri-trophic food chains and exploitative competition have significant negative effects. The additional configurations not found to be significant improve the models, but their individual effects on the food web structure are not significant. Thus, we refrain from making any inference about their effects.

**Figure 3.**
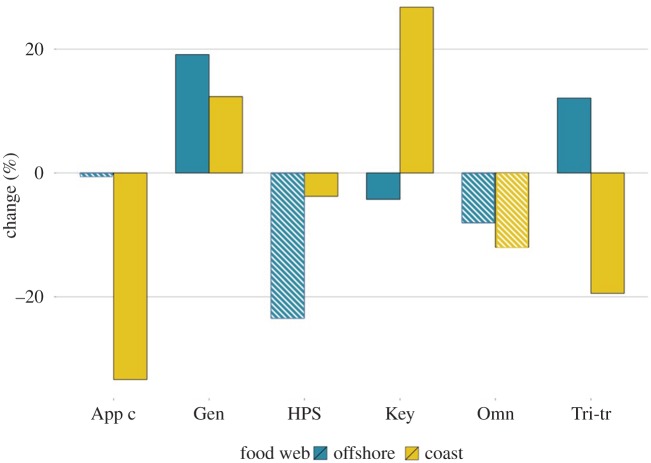
Difference in ERGM configuration parameter estimates from the 1980s to 2000s, i.e. before to after regime shift. The configurations that are not significant (HPS, Omn and in addition App c for offshore) are marked with lighter colour. ‘App c’: apparative competition, ‘Gen’: generalist, ‘HPS': highly predated species, ‘Key’: keystone species, ‘Omn’: omnivory, ‘Tri-tr’: tri-trophic food chain.

Comparing the ERGMs before and after the reported regime shift, we detect clear changes in the coastal region: the parameter estimate for apparent competition has decreased close to 30%, followed by a decrease in the tri-trophic chain by nearly 20% (figure 3). Thus, these processes have seemingly become less important in shaping the coastal food webs. The impact of keystone species and generalists in shaping the structure of the coastal food webs has reversely increased since the 1980s. The changes in the offshore food webs are more modest, although we, in line with what is seen in the coastal region, detect an increase in the generalists. Tri-trophic food chains have increased by 10%, which differs from the coastal system where the direction of change was the opposite.

## 4. Discussion

Our results present the minimal set of processes that give rise to the Baltic Sea food webs. The results indicate that this set of configurations is sufficient to explain the entire food web structure, and that no other processes (i.e. configurations) are taking part in significantly shaping the food webs. In all, the similarity of the set of configurations across all four food webs shows that the coastal and offshore food webs are shaped by similar processes. The results however indicate that the offshore food web is less functionally complex since only three configurations came out as significant in the ERGM (table 2). In comparison, the coastal food web included five significant configurations (table 2).

By examining the detected sets of configurations, we can obtain insights about the community ecology processes that assemble food webs.

The highly predated-species configuration is significant in the coastal food webs. A plausible explanation could be that the coastal food web includes many fish species in comparison to offshore food webs for which the highly predated species was not a significant configuration. All else being equal, if the number of fish species increase whereas the number of prey species in lower trophic levels remains largely the same (essentially the primary consumers), the number of predators per each prey species becomes larger. Thus, it becomes likely that patterns of aggregation emerge in which some specific prey species becomes exploited by a large number of predators. This can explain the significant and positive parameter estimate for the highly predated-species configuration in the fish-species-rich coastal food web. A similar argument can help to explain the estimates for the generalist configuration in the coastal and the offshore food web. Fish species are often found at higher trophic levels, and the higher the level a species occupies, the more opportunities there are to feed on different species (assuming a food web that follows a triangular shape where the number of species is generally lower higher up in the food chain). Therefore, the more fishes in the food web relative to other species, the higher the likelihood that some species will adopt the behaviour of generalist (and a subsequent increase in the estimate for the ERGM generalist configuration). Lastly, the estimate for apparent competition is significant and negative before and after the regime shift in the coastal food web, and never significant in the offshore food web. In interpreting the negative estimate for apparent competition, the generalist configuration needs to be taken into account. Both these configurations capture a process in which predator species prey on multiple prey species. Since the generalist configuration is given a high and positive parameter estimate for the coastal food webs at all times whereas apparent competition is negative, it suggests that the tendency to feed on a comparatively large set of prey is stronger than the tendency to feed on just two or a few more species.

Moving on from the interpretation of sets of configurations to changes in the functioning of the food webs, our results show that the same underlying processes predominantly drive the Baltic food webs before and after the regime shift. This finding indicates no major shifts in the ways species interact at the community level despite changes in species composition, which include nine species in the coastal food web, and additions of three species in the offshore food web and one in coastal food webs. However, the magnitudes of the estimates for some of the configurations in the ERGM have changed, and these changes are most pronounced in the coastal food web. The largest percentile change is the decrease in the apparent competition in the coastal food web, which indicates that predator competition over a set of common prey species is further reduced after the regime shift.

In the offshore food web changes are not especially big and concern generalists, tri-trophic food chains and keystone species. These minor changes could be explained by the increase of intermediate species. Finally, in both the coastal and the offshore food webs, we see an increase in generalism, indicating that processes in which predator species feed on a large number of prey species have become more common after the regime shift.

It seems plausible to assume that an ecosystem-wide regime shift would, if interpreted from a complex systems perspective, result in large changes in the ERGM (parameter estimate change from, e.g. large positive to large negative, new configurations would be needed to explain the observed food web, some previous and significant configurations would no longer be needed, etc.). In addition, if a new regime were the result of biodiversity degradation and other large disturbances, one would assume a simpler food web that could be explained by a smaller number of configurations. None of these changes were however seen in our case, instead we observed remarkable similarities of the food webs before and after the regime shift. Thus, our results give only very limited support for the occurrence of a system-wide regime shift in the Baltic Sea, albeit we acknowledge more research is needed to precisely define what constitute a system-wide regime shift in food web structure. We also wish to emphasize that we are here discussing system-wide regime shift, and our findings do not in any way invalidate previous finding suggesting that the Baltic Sea has undergone a regime shift as seen from the perspective of a more limited number of interacting species.

We suggest that the maintenance of the dominant species interaction processes in the Baltic Sea before and after the reported regime shift is caused by the high connectivity and the absence of compartmentalization in the observed food webs (electronic supplementary material, table S2). Theoretical studies have argued that these structural features allow the species to compensate for local losses by, for example, switching predation to new prey species [36,37]. Again, as seen from a CAS perspective, these adaptive responses would help to maintain the ecological community in a given regime, and could help to explain the fairly small changes in the ERGMs before and after the regime shift. This adaptive capacity might however only be able to absorb exogenous changes until a critical threshold is reached where the system collapses and enters a fundamentally different regime [38]. Our results therefore speculatively suggest that the overall capacity of the ecological community to buffer disturbances (fishing, eutrophication, etc.) could have decreased, and further change/stressors might tip the coastal and offshore ecological communities into fundamentally different regimes on a larger ecosystem level.

Irrespectively of whether a system-wide regime shift has occurred or not, our interpretations of the results outlined above provide evidence that ERGM is able to analytically capture the causal link between the existence of different micro-level species interaction processes and the emergent and observable structure of the entire food web. Further, it seems capable of doing so under the assumption that the actual number of processes that gives rise to complex structures (and high-level phenomena) is often quite low. Indeed, the parsimonious character of ERGM seemingly provides a powerful tool in the search for these core processes, which are often very hard to reveal in complex systems (cf. [3]).

Our study however suffers from several limitations. First, we are aware of uncertainties caused by the applied food web assembly process (described in the electronic supplementary material), and acknowledge that this can affect the modelling results. Second, fitting an ERGM involves lots of manual trial and error since model convergence is challenging, which presents inherent limitations in terms of the number of configuration combinations that can be tested practically. A noted difficulty in ERGM is which criteria to use in selecting the best fitting model [12] (which we avoided here since we used the same set of configurations for all Baltic food webs (but see the electronic supplementary material, table S7 for offshore 2000s alternative model)). Further, although we stress the potential of ERGM in disentangling causes from effects through its ability to facilitate the identification of which specific motifs are needed to explain the emergent structure of the food web, this ability rests on a number of assumptions. First of all, it rests on the assumption that the network is formed and shaped entirely through micro-level processes following a bottom-up approach. Secondly, as with most other statistical models, the modelling results need to be combined with theoretical reasoning and complementing approaches in order to be fully used. A firm assessment of causes and effects in food web dynamics require a mechanistic understanding on the exact nature of these processes. Even though ERGM can help in revealing the importance of, for example, some predator species tendencies to prey on many different prey species in explaining the structure of the food web, it does not however say anything about why these predators do so.

Our study is based on a pure topological analysis of an extended species assemblage, whereas previous Baltic Sea regime shift studies are based on detailed biomass data drawn from a smaller set of species. The differences in the interpretations of the results illustrate that analyses of changes in food web topology and time-series biomass analyses need to be viewed as complementary approaches. Nonetheless, our study demonstrates how the incorporation of ERGM in food web research provides entirely new analytical possibilities in terms of integrating a complex systems perspective with empirical analyses of dynamic food webs.

## 5. Conclusion

This study presents a new analytic approach to reveal underlying processes in complex food webs (with current computing capability, analysing networks of over 1500 nodes, potentially even more, is possible [39]) and test whether they have changed or shifted significantly in the Baltic Sea due to increased anthropogenic activities. Like complexity theory, the applied ERGM framework accommodates the assumption that the system (entire food web structure) emerges from the combined results of multiple micro-level processes playing out simultaneously. This clear distinction between the dependent variable (the observed network) and the independent variables (various micro-level processes described as network configurations) facilities a shift from descriptive to predictive research on what processes give rise to complex food web structures. The analytical ability of ERGM to link the often hard-to-observe species interaction processes with the observable structure of the food web therefore also provides for a better integration of community ecology theory with empirical analyses of complex food webs.

Regime shifts are, in complexity theory, often (but not always) assumed to result from critical changes in the ways the components of the system interact with each other. When contextualizing this general assumption in food webs where the components constitute species and the interaction processes constitute the ways in which species feed on each other, ERGM can provide an important analytical modelling framework contributing to empirical and theoretical research of regime shifts in ecological communities. Our results from analysing the food webs of the Baltic Sea before and after a reported regime shift highlight the importance of considering the entire ecological community in the analysis. We conclude that our results emphasize the importance of future studies to further address the empirically and theoretically challenging question of whether the previously reported regime shift constitutes a system-wide shift or if it only involves a limited but commercially important set of species.

## Supplementary Material

Supporting Information: Regime shifts in marine communities: a complex systems perspective on food web dynamics

## Endnote

1 The abbreviation ERGM is here used both for exponential random graph modeling and exponential random graph models.

## Data accessibility

The food webs studied in this article are available in Dryad depository: http://dx.doi.org/10.5061/dryad.p9f07.
