# Supplementary material for: Regime shifts in marine communities: a complex systems perspective on food web dynamics
Source: Proc Biol Sci. 2016 Feb 24;283(1825):20152569. doi: 10.1098/rspb.2015.2569 (PMC4810827; doi:10.1098/rspb.2015.2569)
Supplement: Supporting Information: Regime shifts in marine communities: a complex systems perspective on food web dynamics [file rspb20152569supp1.docx]

Supporting Information

**Regime shifts in marine communities: a complex systems perspective on food web dynamics**

Johanna Yletyinen, Örjan Bodin, Benjamin Weigel, Marie C. Nordström, Erik Bonsdorff, Thorsten Blenckner

1. Model setup 2

1.1. General Exponential Random Graph Models 2

1.2. Baltic Sea food web data: food web construction 3

2. Baltic Sea ERGM analysis 6

2.1. Baltic Sea ERGM analysis and model fit 6

2.2. Regime shift changes 8

2.3. Descriptive motif representation 8

3. Tables 10

**Table S1**. ERGM network configurations for directed networks without actor attributes

**Table S2**. The number of nodes, ties and nodes per functional groups in the constructed Baltic Sea food webs

**Table S3.** GoF for the four most common motifs ERGMs: offshore food webs

**Table S4**. GoF for the four most common motifs: coastal food webs

**Table S5**. GoF for the offshore Baltic Sea ERGMs

**Table S6.** GoFs for the Baltic Sea coastal ERGMs

**Table S7**. Difference in unnormalised ERGM parameter values from 1980s to 2000s

**Table S8**. Baltic Sea offshore 2000s alternative model

**Table S9**. Results for the Baltic Sea descriptive motif approach analyses

4. Figures 21

**Figure S1a-d.** Baltic Sea food webs used in the study

**Figure S2.** ERGM workflow

**5. SI References 23**

# 1. Model setup

## 1.1. General Exponential Random Graph Models

In social sciences Exponential Random Graph Models (ERGM), also called *p* class of models,* are used for identification of the processes that create and sustain the network [1]. The overall methodological purpose is to search for a well-fitting statistical model for an empirical network by estimating the driving/inhibiting/neutral effect and magnitude of each selected configuration, and evaluating how adequately the resulting model represents empirical data, i.e. the combination of observed network configurations [1]. Based on the network randomization (model specification set by the researcher), ERGM produces an ensemble of networks, forms a representative mean model of the random graphs, and compares the real network to the mean random network. Instead of configuration over/underrepresentation as in prior food web motif studies, the resulting configuration parameters describe the driving or inhibiting tendency for each selected configuration in the process of replicating the entire observed network structure.

Food webs are formed by a multitude of interactions arranged in complex networks. Individual predator-prey feeding interactions accumulate to local processes of traditional community ecology, and further to entire food webs**.** Food webs are thus descriptions of biological communities where many kind of processes are at play at the same time. The basic theoretical assumptions of ERGM are well-suitable for the open, flexible and dynamic nature of food webs: i) network ties self-organize and are at the same time influenced by node attributes and other exogenous factors; ii) patterns within networks can be seen as evidence for on-going structural processes; iii) multiple processes can operate simultaneously; and iv) link formation is locally constructed (cf. [1]). An important feature that ERGM has compared to previous, descriptive configuration approaches is the complex combination of configurations through multiple and nested processes.

The choice of which configurations to include in ERGM model specification is based on theories relevant to the research question in hand, and in essence states which combinations of processes are considered important in the formation of network structure. ERGM provides a combination of 32 configurations in a model (Table S1). For solid conclusions on basic mechanisms giving rise to the network structure both higher- and lower-order configurations must be observed (Goodness of Fit (GoF) procedure), as a high occurrence of a certain configuration may actually originate from a high occurrence of simpler configurations [1].

If the produced model is sufficient to explain a network feature, the corresponding configuration in the empirical data is not extreme in the distribution of the random graphs. The model fit is signified by t-ratio, a value given by ERGM for each configuration. To know whether we have a good model fit between simulated statistics and observed statistics we followed Lusher *et al*. [1]: we defined a model fit as a GoF with t-ratios for all configurations below 2 and for the configurations used in model specification between 0.1 and -0.1. (with an exception of small difference). Model fit means in practice that the combinations of chosen configurations is a good representation of how the observed network could have formed [1]. In practice, the driving and inhibiting configurations needed to reconstruct the Baltic Sea food webs are the processes that as a set, without the need for any other configurations/processes, are able to give rise to the entire Baltic Sea food web structures.

## 1.2. Baltic Sea food web data: food web construction

The selection of species to be included in the Baltic Sea food webs (Figure S1a-d) was based on their relative biomasses within their functional groups. The criteria for the inclusion of species into the constructed networks are based on the following rules:

**Phytoplankton**: Pooled into one functional group (node). The biomass-based criterion is not applicable to the phytoplankton. Including phytoplankton as individual species would have resulted in extremely high number of phytoplankton nodes, resulting in biased network complexity on one trophic level.

**Microalgae, detritus and bacteria:** One node each. Microalgae are included in coastal food webs as they form a substantial food source for the zoobenthos.

**Zooplankton:** Zooplankton species above 5% of the total biomass were used based on the sampling data and calculations by Finnish Institute of Marine Research [2] for the offshore, and specific sampling data for the coastal area.

**Zoobenthos:** All species with biomass-contributions above 5% of the total benthic biomass were included in all food webs, based on specific sampling both in the offshore and coastal regions.

**Fish:** For coastal food webs the selection of species resulted from fish surveys, and in uncertain cases (e.g. lack of data) ecologically important species were added based on expert opinion. For the offshore food webs, expert opinions were used for selecting which species to include for each decade, as data for non-commercial pelagic fish biomass fluctuations is not available. Due to the early stage importance of cod on the zooplankton – sprat – cod interactions [3], cod in the offshore food web is represented by two nodes: adult cod and cod larvae.

**Mammals**: The inclusion of seals into the 2000s food webs was based on expert opinion and literature on the increase of seal populations in the Baltic Sea during the last two decades [4,5].

**Birds**: Although we are aware of sea bird predation on a number of Baltic Sea species, they are not included in the food webs analysed here. In our study regions the occurrence of birds is patchy and highly seasonal, and knowledge on the diet of some important species and life stages is very limited. Including birds would have included addition of coastal – offshore linkage for the offshore species, as most birds forage on the open sea but nest on the land.

Predator-prey interactions were based on existing sampling data when available, or secondarily on literature studies and expert opinions. During the food web collection and in the finalization phase, expert opinions were asked also for evaluating how realistic for the Baltic Sea each food web entity was in their species selection and prey-predation relationships. Table S2 presents the basic features of the constructed food webs.

**1.3. Baltic Sea late-1980s regime shift**

In the late-1980s several, almost synchronous marine regime shifts were reported for the northern hemisphere, including the Baltic Sea [6]. The central Baltic Sea food web is relatively simple and the fish community is dominated by three species: cod, sprat and herring. A period of ecological stress and anthropogenic impacts in the late-1980s and early-1990s caused the biotic part of the central Baltic Sea to shift to an altered state of reduced cod productivity [7,8]. It is assumed that the regime shift in the open sea was induced by a combination of interacting effects, firstly from eutrophication and fishing pressure and secondly by environmental drivers, such as increasing temperature and decreasing salinity [7,3]. A trophic cascade occurred when the shift from cod to clupeids took place in combination with climate-driven changes [7–9]. Because of reduced predation by cod and environmental conditions favorable for sprat, the sprat stocks increased largely [7,9,10]. Changes in zooplankton composition influenced prey availability for both cod and sprat and maybe promoted algal blooms on the Baltic Sea [7–9]. Predator-prey feedback loops thus appear to be stabilizing the system [11]. The central Baltic Sea regime shift has been relatively well described in numerous studies, focusing mainly on shifts in zooplankton composition and inverse changes of cod and sprat abundances (and biomasses) (e.g. [3,12]), enforcing altered tropho-dynamic pathways (e.g. [13] ).

Coastal areas, on the other hand, still remain relatively unknown regarding regime shift analysis. However, some studies show that coastal communities have undergone major species compositional changes at coinciding years as well [14,15]. The benthic components at the Åland Islands show a gradual change in community composition over time, and the magnitude of change seemed pronounced in times coinciding with the suggested time frame of the late Baltic Sea regime shift, and supports its reported potential to impact all trophic levels [15]. This corresponds well with Rousi *et al.* [14] who found in area close by (entrance of the Gulf of Finland) significant shifts in the long-term zoobenthos composition data. Also changes in the fish abundance has been reported during the same period (late-1980s), mainly caused by changes in Secci depth and temperature [16]. So overall, it seems that the drivers are similar in the case of the offshore and coastal regime shift but the food web responses to these drivers are different. In general, it has been shown that many ecosystems globally change drastically in the late 1980s due to changes in temperature [17]. The impact of anthropogenically induced eutrophication, originating from land, as well as climate change induced shifts in hydrographical conditions are more pronounced in the comparably shallow coastal areas than in the larger and deeper water bodies offshore [15,16]. This suggests that regime shifts previously described for the open Baltic Sea, could also be found in their adjacent coastal areas, influencing community compositions and food-web dynamics of these highly productive zones.

# 2. Baltic Sea ERGM analysis

## 2.1. Baltic Sea ERGM analysis and model fit

Our Baltic Sea empirical analysis aimed to find the configurations that give rise to the network structure of the Baltic Sea food webs so that none, or at least as few as possible, of the model network features (i.e. 32 ERGM configurations in GoF) significantly differ from the features of empirical food webs. We examined a set of 32 configurations provided by the ERGM modelling tools (Table S1). The following analyses were performed for each food web separately. All ERGM analyses are performed with MPNET software (The University of Melbourne. http://sna.unimelb.edu.au/PNet).

Previous research has shown that tri-trophic chain, omnivory, apparent competition and exploitative competition represent 95% of the three-species motifs observed empirically [18,19]. The first analysis tested if these four motifs could explain the structure of the marine food webs. We specified ERGM with the number of nodes and links and tri-trophic chain, omnivory, apparent competition and exploitative competition (ERGM configurations 2Path, Transitive Triad, In2Star, Out2Star, and Arc to control network density). The features of the observed networks that significantly differ in GoF statistics from the four motifs’ random models originate from other processes than those represented by the four motifs. If such configurations occur, it is assumed that the food webs cannot be arisen solely from the four most commonly occurring food web motifs. The results presented in Tables S3 and S4 show that the four motifs’ model specification produced a fitted model for the offshore 1980s food web, but struggled with the model fit for the offshore 2000s, and produced extreme values for the coastal food webs. These results signify that the four motifs are not adequate on their own to structurally explain the observed Baltic Sea food webs.

In the second part of the analysis, we extended our search for explanatory configurations beyond the four most common food web motifs. We explored the ERGMs to find the *minimal* set of configurations that are able to replicate the features of the observed Baltic Sea food webs. GoF for the resulting models are presented in Tables S5 and S6. This analysis utilizes the ERGM feature that permits inferences for the testing of hypotheses. Namely, in the case of no model convergence, the researcher makes a choice of additional configuration or configurations to see if they assist model fit. The selection of configurations (model specification) is modified until the model convergences.

The following procedure explains the model selection process we applied on the Baltic Sea food webs in the second part of the analysis, demonstrating the empirical character of the ERGM estimation analysis with MPNET (Figure S2). We used alternate configurations as these higher-order configurations are better able to model denser regions of networks that occur in many observed networks [1,20].

i) We started with the simplest way of building an ERGM by specifying the model only with the number of ties (Arc) [1]. For the next estimation, the ERGM was specified with those alternating configurations that had the most extreme t-ratios in the Arc-specified ERGM GoF.

ii) The basic idea of ERGM is to assess how well the model manages to capture network features that were not explicitly modeled. This is done continuously during the model specification.

- We examined changes in t-ratios and Mahalanobis distance (MD) and gradually added configurations for those network features that needed improvement (see definition for model fit below) in the model. One added configuration can improve several t-ratios.

- If the MD had dropped significantly but t-ratios were not sufficiently low, we added one more configuration to the model specification.

- If the model needed improvement but did not show which configuration would improve the model fit, we added a configuration with the most extreme t-ratio in the Arc-specified GoF, if not yet in the current model and examined the resulting changes.

iii) With every converged model, we tested model stability by re-running the model to see that the parameter statistics and significant configurations remain the same, and no extreme t-ratios emerge. If the model was not stable, it was rejected.

iv) When the model fit emerged, we removed the configurations in the reversed order to test their effect. Neutral configurations were removed.

v) In the end we tested specifying each model only with their significant configurations (i.e. a configuration with an absolute value more than twice its standard error in parameter estimates [1]) to study the effect of insignificant configuration. If an insignificant configuration improved structural details of the model, it was kept in the specification.

vi) In case of several model fits, we selected the model with the lowest MD.

## 2.2. Regime shift changes

Lastly, we studied the potential regime shift changes. Parameter estimations needed to specify each Baltic Sea ERGM were collected and the difference from one regime to another was calculated as the percentage difference in parameter values from the 1980s to 2000 (Table S7). All four food webs analysed in this study are constructed by the authors, following the same network boundary setting principles. Offshore and coastal food webs are compared separately. For these reasons and due to the same set of configuration for all food webs we consider the regional comparison well-grounded, aware of the cross-system consideration guidelines [1]. The same comparison was made for the descriptive motif representation results by using z-scores, which indicate the significance of deviations of the real network from the null hypothesis (i.e. random model).

### 2.3. Descriptive motif representation

For comparative purposes we also assessed the fractional changes of tri-trophic food chain, omnivory, apparent competition and exploitative competition using the previous, descriptive motif-based approach, which examines the significance of a particular motif (configuration) by comparing the motif over/underrepresentation from observed and model generated random food webs [21,22]. In this approach the random models are specified with number of prey, number of predators, number of single links (A->B), number of double links (A->B, B->A) and whether or not a species is cannibal. In our food webs cannibalism is neglected. Single, double and cannibal links are treated separately. This approach does not count the motif instances similarly to ERGM and includes only 3 nodes’ motifs (n = 13) into analysis.

Using this method, we only find small changes in the coastal region: less than 10 per cent decrease in tri-trophic chains, exploitative competition and apparent competition, and less than 10 per cent increase in omnivory. However, for the offshore region the descriptive motif approach detects over 40 per cent increase in omnivory, 30 and 40 per cent decrease in exploitative and apparent competition configurations, respectively, and over 20 per cent decrease in tri-trophic food chain configurations. Notice, however, that the descriptive motif approach quantifies the frequency of motifs in the food web, whereas the ERGM examines which substructures, as a set, give rise to the observed food web structure. Thus, the results of these two approaches are not comparable due to different purpose and due to the fact that in ERGM the parameter estimate for each configuration is affected by the presence of other configurations in model specification.

The results of the descriptive motif analysis (Table S9) give a practical example of how ERGM extends the motif research on food webs. Whereas the descriptive motif approach identifies the mechanisms responsible for the observed food web structure based on whether their representation in network significantly differs from null hypothesis (random model ensemble), ERGM provides a statistical tool for testing which set of configurations give rise to the observed food web structure. ERGM examines also configurations not selected in model specification (GoF procedure) to study whether the selected configurations can explain the entire food web structure, in which case they can be considered the structures responsible for he observed food web structure. In addition, ERGM takes into account secondary effects of certain configurations being more or less occurring (i.e. “motif embeddedness”), a feature that has often been lacking in food web motif analysis.

# 3. Tables

**Table S1.** ERGM network configurations for directed networks without actor attributes [23]


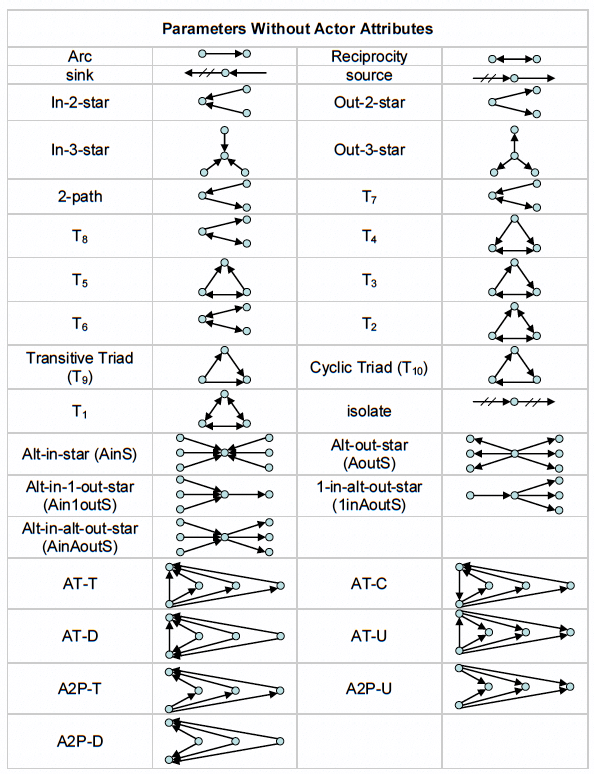


Table S2. The number of nodes, ties and nodes per functional groups in the constructed Baltic Sea food webs. The connectance and linkage density are calculated as in Pimm *et al.* [24]

|  | OFFSHORE BALTIC SEA  FOOD WEBS | | COASTAL BALTIC SEA  FOOD WEBS | |
| --- | --- | --- | --- | --- |
|  | **1980s** | **2000s** | **1980s** | **2000s** |
| Nodes | 28 | 31 | 33 | 34 |
| Ties | 102 | 115 | 184 | 192 |
| Connectance | 0.27 | 0.25 | 0.35 | 0.34 |
| Linkage density | 3.64 | 3.71 | 5.58 | 5.65 |
| Number of nodes per functional group |  | | | |
| Phytoplankton | 1 | 1 | 1 | 1 |
| Bacteria | 1 | 1 | 1 | 1 |
| Detritus | 1 | 1 | 1 | 1 |
| Microalgae | - | - | 1 | 1 |
| Zooplankton | 14 | 14 | 11 | 10 |
| Benthic species | 5 | 7 | 5 | 6 |
| Fish | 6 | 6 | 13 | 13 |
| Marine mammals | - | 1 | - | 1 |

Table S3. GoF for the four most common motifs ERGMs: offshore food webs. Model specification marked with bold letters.

|  | OFFSHORE 1980s | | | | OFFSHORE 2000s | | | |
| --- | --- | --- | --- | --- | --- | --- | --- | --- |
| Network configuration | Observed food web | Random network, mean | St. err. | T-ratio | Observed food web | Random network, mean | St. err. | T-ratio |
| **ArcA** | **102** | **102.014** | **17.064** | **-0.001** | **115** | **112.359** | **16.405** | **0.161** |
| ReciprocityA | 2 | 3.333 | 1.895 | -0.704 | 0 | 3.532 | 1.964 | -1.798 |
| **In2StarA** | **326** | **329.35** | **167.761** | **-0.02** | **367** | **338.751** | **144.738** | **0.195** |
| **Out2StarA** | **255** | **247.429** | **94.164** | **0.08** | **286** | **272.754** | **96.333** | **0.138** |
| In3StarA | 904 | 1122.008 | 1065.412 | -0.205 | 984 | 1114.727 | 991.348 | -0.132 |
| Out3StarA | 589 | 510.477 | 375.918 | 0.209 | 630 | 583.649 | 433.97 | 0.107 |
| **TwoPathA** | **227** | **228.427** | **62.326** | **-0.023** | **273** | **264.332** | **67.489** | **0.128** |
| **Transitive-TriadA** | **71** | **69.794** | **35.149** | **0.034** | **76** | **69.119** | **33.64** | **0.205** |
| Cyclic-TriadA | 0 | 4.319 | 3.043 | -1.42 | 0 | 4.645 | 3.054 | -1.521 |
| T1A | 0 | 0.002 | 0.045 | -0.045 | 0 | 0 | 0 | NaN |
| T2A | 0 | 0.193 | 0.546 | -0.354 | 0 | 0.133 | 0.379 | -0.351 |
| xT3A | 0 | 2.023 | 2.229 | -0.908 | 0 | 1.86 | 2.145 | -0.867 |
| T4A | 2 | 1.846 | 2.176 | 0.071 | 0 | 1.772 | 2.048 | -0.865 |
| T5A | 4 | 2.291 | 2.304 | 0.742 | 0 | 2.065 | 2.167 | -0.953 |
| T6A | 1 | 0.925 | 1.393 | 0.054 | 0 | 0.855 | 1.277 | -0.669 |
| T7A | 10 | 24.987 | 18.175 | -0.825 | 0 | 25.678 | 17.891 | -1.435 |
| T8A | 16 | 23.746 | 15.886 | -0.488 | 0 | 24.913 | 16.854 | -1.478 |
| SinkA | 3 | 2.543 | 1.389 | 0.329 | 3 | 2.615 | 1.467 | 0.263 |
| SourceA | 3 | 3.388 | 1.837 | -0.211 | 3 | 3.202 | 1.664 | -0.121 |
| IsolateA | 0 | 0.033 | 0.184 | -0.179 | 0 | 0.04 | 0.201 | -0.199 |
| AinSA | 125.6045 | 123.8371 | 32.53 | 0.054 | 143.2139 | 133.7595 | 30.172 | 0.313 |
| AoutSA | 118.3792 | 118.7844 | 30.664 | -0.013 | 135.0042 | 129.9656 | 29.31 | 0.172 |
| AinSA2 | 125.6045 | 123.8371 | 32.53 | 0.054 | 143.2139 | 133.7595 | 30.172 | 0.313 |
| AoutSA2 | 118.3792 | 118.7844 | 30.664 | -0.013 | 135.0042 | 129.9656 | 29.31 | 0.172 |
| AinAoutSA | 54.975 | 56.9855 | 7.941 | -0.253 | 61.213 | 65.5808 | 7.987 | -0.547 |
| ATA-T | 43.3589 | 52.0176 | 22.285 | -0.389 | 46.8589 | 52.3651 | 21.074 | -0.261 |
| ATA-C | 0 | 12.0189 | 8.056 | -1.492 | 0 | 13.0127 | 8.102 | -1.606 |
| ATA-D | 53.375 | 51.0456 | 21.204 | 0.11 | 54.375 | 52.1509 | 20.804 | 0.107 |
| ATA-U | 59.75 | 53.3691 | 21.847 | 0.292 | 63.5 | 55.0073 | 22.753 | 0.373 |
| A2PA-T | 145.9585 | 192.8422 | 47.445 | -0.988 | 171.7866 | 226.6727 | 52.197 | -1.052 |
| A2PA-D | 187.1582 | 178.7694 | 55.537 | 0.151 | 203.0332 | 208.617 | 61.273 | -0.091 |
| A2PA-U | 230.4688 | 241.1994 | 89.508 | -0.12 | 255.4375 | 264.4839 | 95.348 | -0.095 |
| stddev_  indegreeA | 3.7635 | 3.5719 | 1.125 | 0.17 | 3.7523 | 3.3967 | 0.963 | 0.369 |
| skew_  indegreeA | 1.3058 | 1.4658 | 0.761 | -0.21 | 1.1831 | 1.5212 | 0.85 | -0.398 |
| stddev_outdegreeA | 2.9841 | 2.7461 | 0.644 | 0.37 | 2.9461 | 2.7704 | 0.624 | 0.282 |
| skew_out  degreeA | 1.5925 | 0.846 | 0.639 | 1.168 | 1.4262 | 0.9808 | 0.621 | 0.717 |
| clusteringA_tm | 0.3128 | 0.2982 | 0.115 | 0.127 | 0.2784 | 0.2551 | 0.089 | 0.262 |
| clusteringA_cm | 0 | 0.0546 | 0.033 | -1.676 | 0 | 0.0506 | 0.027 | -1.855 |
| clusteringA_ti | 0.1089 | 0.1069 | 0.026 | 0.077 | 0.1035 | 0.1014 | 0.024 | 0.093 |
| clusteringA_to | 0.1392 | 0.1433 | 0.025 | -0.168 | 0.1329 | 0.1249 | 0.038 | 0.211 |
|  | Mahalanobis distance = 1983 | | | | Mahalanobis distance = 3423 | | | |

Table S4. GoF for the four most common motifs: coastal food webs. Model specification marked with bold letters.

|  | COAST 1980s | | | | | COAST 2000s | | | |
| --- | --- | --- | --- | --- | --- | --- | --- | --- | --- |
| Network configuration | Observed food web | Random network, mean | St. err. | | T-ratio | Empirical food web | Random network, mean | St. err. | T-ratio |
| **ArcA** | **184** | **194.468** | **36.023** | | **-0.291** | **192** | **185.243** | **24.581** | **0.275** |
| ReciprocityA | 0 | 6.691 | 2.914 | | -2.296 | 0 | 7.561 | 2.753 | -2.746 |
| **In2StarA** | **858** | **1027.723** | **550.423** | | **-0.308** | **841** | **735.996** | **233.302** | **0.45** |
| **Out2StarA** | **738** | **809.423** | **334.882** | | **-0.213** | **794** | **717.014** | **248.667** | **0.31** |
| In3StarA | 2825 | 4786.118 | 3773.071 | | -0.52 | 2570 | 2568.473 | 1427.822 | 0.001 |
| Out3StarA | 2113 | 2680.897 | 1627.202 | | -0.349 | 2431 | 2390.558 | 1499.397 | 0.027 |
| **TwoPathA** | **584** | **585.622** | **128.634** | | **-0.013** | **630** | **637.471** | **127.003** | **-0.059** |
| **Transitive-TriadA** | **222** | **237.402** | **84.231** | | **-0.183** | **226** | **208.727** | **76.743** | **0.225** |
| Cyclic-TriadA | 0 | 13.14 | 7.176 | | -1.831 | 0 | 15.615 | 6.485 | -2.408 |
| T1A | 0 | 0.005 | 0.071 | | -0.071 | 0 | 0.006 | 0.077 | -0.078 |
| T2A | 0 | 0.789 | 1.256 | | -0.628 | 0 | 0.879 | 1.208 | -0.728 |
| T3A | 0 | 8.027 | 5.702 | | -1.408 | 0 | 8.646 | 5.321 | -1.625 |
| T4A | 0 | 7.59 | 5.669 | | -1.339 | 0 | 8.197 | 5.803 | -1.413 |
| T5A | 0 | 8.782 | 6.513 | | -1.348 | 0 | 7.885 | 5.211 | -1.513 |
| T6A | 0 | 3.124 | 3.343 | | -0.935 | 0 | 3.549 | 3.25 | -1.092 |
| T7A | 0 | 81.112 | 39.315 | | -2.063 | 0 | 83.298 | 38.795 | -2.147 |
| T8A | 0 | 74.383 | 37.945 | | -1.96 | 0 | 80.891 | 36.01 | -2.246 |
| SinkA | 4 | 2.421 | 2.001 | | 0.789 | 3 | 1.727 | 1.291 | 0.986 |
| SourceA | 4 | 2.821 | 2.345 | | 0.503 | 4 | 1.818 | 1.219 | 1.791 |
| IsolateA | 0 | 0.001 | 0.032 | | -0.032 | 0 | 0.003 | 0.055 | -0.055 |
| AinSA | 274.5898 | 284.2679 | 78.689 | | -0.123 | 284.3495 | 256.6649 | 48.213 | 0.574 |
| AoutSA | 266.5697 | 279.4266 | 75.452 | | -0.17 | 275.6132 | 255.874 | 48.859 | 0.404 |
| AinSA2 | 274.5898 | 284.2679 | 78.689 | | -0.123 | 284.3495 | 256.6649 | 48.213 | 0.574 |
| AoutSA2 | 266.5697 | 279.4266 | 75.452 | | -0.17 | 275.6132 | 255.874 | 48.859 | 0.404 |
| AinAoutSA | 65.9241 | 82.7713 | 15.296 | | -1.101 | 74.6565 | 93.1841 | 7.449 | -2.487 |
| ATA-T | 138.8115 | 157.3389 | 50.549 | | -0.367 | 142.8652 | 139.4357 | 41.677 | 0.082 |
| ATA-C | 0 | 34.2889 | 17.515 | | -1.958 | 0 | 40.9517 | 15.632 | -2.62 |
| ATA-D | 98.8506 | 145.3142 | 39.604 | | -1.173 | 103.7285 | 135.6297 | 36.939 | -0.864 |
| ATA-U | 130.6299 | 139.3307 | 35.945 | | -0.242 | 135.4907 | 137.5829 | 37.992 | -0.055 |
| A2PA-T | 314.0573 | 446.0938 | 84.055 | | -1.571 | 332.051 | 492.6976 | 81.714 | -1.966 |
| A2PA-D | 327.2751 | 394.3358 | 92.348 | | -0.726 | 357.3008 | 438.5859 | 95.198 | -0.854 |
| A2PA-U | 398.2363 | 480.2052 | 103.286 | -0.794 | | 411.2461 | 454.979 | 94.442 | -0.463 |
| stddev_indegreeA | 5.2263 | 5.4511 | 1.885 | -0.119 | | 4.8921 | 4.272 | 0.92 | 0.674 |
| skew_indegreeA | 0.412 | 1.0759 | 0.599 | | -1.109 | 0.2903 | 1.048 | 0.639 | -1.186 |
| stddev_outdegreeA | 4.4513 | 4.2882 | 1.112 | | 0.147 | 4.5917 | 4.1186 | 0.992 | 0.477 |
| skew_outdegreeA | 0.331 | 0.6096 | 0.76 | | -0.367 | 0.4959 | 0.9398 | 0.524 | -0.847 |
| clusteringA_tm | 0.3801 | 0.4106 | 0.139 | | -0.218 | 0.3587 | 0.3215 | 0.081 | 0.459 |
| clusteringA_cm | 0 | 0.0647 | 0.029 | | -2.269 | 0 | 0.0724 | 0.024 | -3.074 |
| clusteringA_ti | 0.1294 | 0.1292 | 0.038 | | 0.005 | 0.1344 | 0.1409 | 0.025 | -0.263 |
| clusteringA_to | 0.1504 | 0.1531 | 0.036 | | -0.074 | 0.1423 | 0.146 | 0.027 | -0.136 |
|  | Mahalanobis distance = 5433 | | | | | Mahalanobis distance = 4493 | | | |

Table S5. GoF for the offshore Baltic Sea ERGMs. Model specification marked with bold letters.

|  | OFFSHORE 1980s | | | | | OFFSHORE 2000s | | | |
| --- | --- | --- | --- | --- | --- | --- | --- | --- | --- |
| Network configuration | Observed food web | Random network, mean | St. err. | | T-ratio | Observed food web | Random network, mean | St. err. | T-ratio |
| **ArcA** | **102** | **102.819** | **13.595** | | **-0.06** | **115** | **114.663** | **12.057** | **0.028** |
| ReciprocityA | 2 | 2.159 | 1.57 | | -0.101 | 0 | 2.279 | 1.469 | -1.551 |
| In2StarA | 326 | 304.786 | 104.308 | | 0.203 | 367 | 322.26 | 80.658 | 0.555 |
| Out2StarA | 255 | 258.001 | 85.371 | | -0.035 | 286 | 308.072 | 85.002 | -0.26 |
| In3StarA | 904 | 783.117 | 460.736 | | 0.262 | 984 | 733.607 | 329.836 | 0.759 |
| Out3StarA | 589 | 539.806 | 300.301 | | 0.164 | 630 | 747.99 | 374.557 | -0.315 |
| TwoPathA | 227 | 191.735 | 38.336 | | 0.92 | 273 | 219.462 | 38.547 | 1.389 |
| Transitive-TriadA | 71 | 75.614 | 32.805 | | -0.141 | 76 | 77.461 | 26.624 | -0.055 |
| Cyclic-TriadA | 0 | 0.777 | 1.109 | | -0.701 | 0 | 0.934 | 1.107 | -0.844 |
| T1A | 0 | 0.001 | 0.032 | | -0.032 | 0 | 0 | 0 | NaN |
| T2A | 0 | 0.064 | 0.319 | | -0.2 | 0 | 0.058 | 0.246 | -0.236 |
| xT3A | 0 | 0.428 | 0.876 | | -0.489 | 0 | 0.458 | 0.838 | -0.547 |
| T4A | 2 | 2.264 | 2.74 | | -0.096 | 0 | 2.854 | 2.809 | -1.016 |
| T5A | 4 | 1.158 | 1.449 | | 1.962 | 0 | 0.864 | 1.225 | -0.705 |
| T6A | 1 | 0.266 | 0.601 | | 1.221 | 0 | 0.236 | 0.546 | -0.432 |
| T7A | 10 | 15.2 | 12.79 | | -0.407 | 0 | 19.401 | 14.016 | -1.384 |
| T8A | 16 | 9.723 | 8.717 | | 0.72 | 0 | 8.785 | 7.739 | -1.135 |
| SinkA | 3 | 1.713 | 0.955 | | 1.347 | 3 | 1.57 | 0.997 | 1.435 |
| SourceA | 3 | 4.162 | 1.427 | | -0.814 | 3 | 5.04 | 1.509 | -1.352 |
| IsolateA | 0 | 0.133 | 0.362 | | -0.367 | 0 | 0.189 | 0.455 | -0.415 |
| **AinSA** | **125.6045** | **127.2188** | **26.557** | | **-0.061** | **143.2139** | **142.3413** | **22.284** | **0.039** |
| **AoutSA** | **118.3792** | **119.907** | **25.386** | | **-0.06** | **135.0042** | **134.3419** | **22.493** | **0.029** |
| AinSA2 | 125.6045 | 127.2188 | 26.557 | | -0.061 | 143.2139 | 142.3413 | 22.284 | 0.039 |
| AoutSA2 | 118.3792 | 119.907 | 25.386 | -0.06 | | 135.0042 | 134.3419 | 22.493 | 0.029 |
| **AinAoutSA** | **54.975** | **54.9446** | **4.858** | **0.006** | | **61.213** | **61.353** | **5.483** | **-0.026** |
| ATA-T | 43.3589 | 51.8029 | 16.454 | -0.513 | | 46.8589 | 55.5516 | 14.712 | -0.591 |
| ATA-C | 0 | 2.264 | 3.159 | -0.717 | | 0 | 2.717 | 3.149 | -0.863 |
| ATA-D | 53.375 | 52.8181 | 16.749 | 0.033 | | 54.375 | 57.0245 | 15.588 | -0.17 |
| ATA-U | **59.75** | **60.4941** | **22.158** | **-0.034** | | **63.5** | **62.6398** | **18.056** | **0.048** |
| A2PA-T | **145.9585** | **145.5821** | **20.282** | **0.019** | | **171.7866** | **173.1071** | **24.237** | **-0.054** |
| A2PA-D | **187.1582** | **189.7945** | **44.805** | **-0.059** | | 203.0332 | 235.9584 | 51.83 | -0.635 |
| A2PA-U | **230.4688** | **233.7899** | **60.035** | **-0.055** | | **255.4375** | **253.1097** | **49.193** | **0.047** |
| stddev_  indegreeA | 3.7635 | 3.4253 | 0.651 | 0.519 | | 3.7523 | 3.2864 | 0.461 | 1.012 |
| skew_  indegreeA | 1.3058 | 1.0602 | 0.348 | 0.706 | | 1.1831 | 0.8685 | 0.314 | 1.002 |
| stddev_outdegreeA | 2.9841 | 2.8971 | 0.546 | 0.159 | | 2.9461 | 3.1296 | 0.522 | -0.352 |
| skew_out  degreeA | 1.5925 | 0.9377 | 0.348 | 1.882 | | 1.4262 | 1.2726 | 0.416 | 0.369 |
| clusteringA_tm | 0.3128 | 0.3822 | 0.106 | -0.657 | | 0.2784 | 0.3473 | 0.078 | -0.882 |
| clusteringA_cm | 0 | 0.0129 | 0.018 | -0.705 | | 0 | 0.013 | 0.016 | -0.832 |
| clusteringA_ti | 0.1089 | 0.1213 | 0.018 | -0.672 | | 0.1035 | 0.1184 | 0.018 | -0.828 |
| clusteringA_to | 0.1392 | 0.1433 | 0.025 | -0.168 | | 0.1329 | 0.1246 | 0.018 | 0.448 |
|  | Mahalanobis distance = 2273 | | | | | Mahalanobis distance = 3273 | | | |

Table S6. GoFs for the Baltic Sea coastal ERGMs. Model specification marked with bold letters.

|  | COAST 1980s | | | | | COAST 2000s | | | |
| --- | --- | --- | --- | --- | --- | --- | --- | --- | --- |
| Network configuration | Empirical food web | Random network, mean | St. err. | | T-ratio | Empirical food web | Random network, mean | St. err. | T-ratio |
| **ArcA** | **184** | **185.612** | **15.478** | | **-0.104** | **192** | **191.171** | **17.494** | **0.047** |
| ReciprocityA | 0 | 2.651 | 1.767 | | -1.501 | 0 | 3.226 | 1.978 | -1.631 |
| In2StarA | 858 | 760.941 | 121.724 | | 0.797 | 841 | 752.565 | 141.349 | 0.626 |
| Out2StarA | 738 | 827.998 | 149.723 | | -0.601 | 794 | 888.007 | 178.732 | -0.526 |
| In3StarA | 2825 | 2122.654 | 536.746 | | 1.309 | 2570 | 1999.384 | 596.081 | 0.957 |
| Out3StarA | 2113 | 2843.064 | 803.033 | | -0.909 | 2431 | 3332.757 | 1019.27 | -0.885 |
| TwoPathA | 584 | 452.191 | 89.486 | | 1.473 | 630 | 485.642 | 83.945 | 1.72 |
| Transitive-TriadA | 222 | 209.39 | 53.822 | | 0.234 | 226 | 211.33 | 59.871 | 0.245 |
| Cyclic-TriadA | 0 | 1.911 | 1.92 | | -0.995 | 0 | 2.333 | 2.022 | -1.154 |
| T1A | 0 | 0.001 | 0.032 | | -0.032 | 0 | 0 | 0 | NaN |
| T2A | 0 | 0.112 | 0.444 | | -0.252 | 0 | 0.08 | 0.309 | -0.259 |
| xT3A | 0 | 0.993 | 1.467 | -0.677 | | 0 | 1.034 | 1.392 | -0.743 |
| T4A | 0 | 6.138 | 4.734 | -1.297 | | 0 | 6.785 | 4.747 | -1.429 |
| T5A | 0 | 1.481 | 2.327 | | -0.637 | 0 | 1.182 | 2.1 | -0.563 |
| T6A | 0 | 0.569 | 1.054 | | -0.54 | 0 | 0.688 | 1.165 | -0.591 |
| T7A | 0 | 36.445 | 24.964 | | -1.46 | 0 | 44.411 | 26.981 | -1.646 |
| T8A | 0 | 15.531 | 13.88 | | -1.119 | 0 | 15.895 | 13.135 | -1.21 |
| SinkA | 4 | 2.798 | 1.514 | | 0.794 | 3 | 1.3 | 1.14 | 1.491 |
| SourceA | 4 | 7.222 | 1.713 | | -1.881 | 4 | 6.479 | 1.796 | -1.38 |
| IsolateA | 0 | 0.519 | 0.721 | | -0.72 | 0 | 0.698 | 0.829 | -0.842 |
| **AinSA** | **274.5898** | **277.3909** | **28.769** | | **-0.097** | **284.3495** | **282.8999** | **33.359** | **0.043** |
| **AoutSA** | **266.5697** | **269.2022** | **30.171** | | **-0.087** | **275.6132** | **274.2893** | **34.352** | **0.039** |
| AinSA2 | 274.5898 | 277.3909 | 28.769 | | -0.097 | 284.3495 | 282.8999 | 33.359 | 0.043 |
| AoutSA2 | 266.5697 | 269.2022 | 30.171 | | -0.087 | 275.6132 | 274.2893 | 34.352 | 0.039 |
| **AinAoutSA** | **65.9241** | **66.2242** | **7.921** | | **-0.038** | **74.6565** | **74.6392** | **7.574** | **0.002** |
| ATA-T | 138.8115 | 138.4309 | 26.711 | | 0.014 | 142.8652 | 136.4423 | 28.35 | 0.227 |
| ATA-C | 0 | 5.4055 | 5.241 | | -1.031 | 0 | 6.6213 | 5.57 | -1.189 |
| ATA-D | 98.8506 | 120.0797 | 26.448 | | -0.803 | 103.7285 | 126.1366 | 29.484 | -0.76 |
| **ATA-U** | **130.6299** | **132.7763** | **25.038** | | **-0.086** | **135.4907** | **136.2735** | **26.924** | **-0.029** |
| **A2PA-T** | **314.0573** | **315.3871** | **50.839** | | **-0.026** | **332.051** | **332.6998** | **45.715** | **-0.014** |
| A2PA-D | 327.2751 | 420.6986 | 55.032 | | -1.698 | 357.3008 | 466.7823 | 63.819 | -1.716 |
| **A2PA-U** | **398.2363** | **403.2118** | **40.371** | | **-0.123** | **411.2461** | **409.0507** | **43.459** | **0.051** |
| stddev_indegreeA | 5.2263 | 4.5118 | 0.388 | | 1.839 | 4.8921 | 4.2847 | 0.447 | 1.36 |
| skew_indegreeA | 0.412 | 0.0929 | 0.164 | | 1.944 | 0.2903 | 0.0046 | 0.163 | 1.751 |
| stddev_outdegreeA | 4.4513 | 4.9437 | 0.509 | | -0.968 | 4.5917 | 5.143 | 0.562 | -0.982 |
| skew_outdegreeA | 0.331 | 0.6557 | 0.191 | | -1.698 | 0.4959 | 0.8824 | 0.216 | -1.785 |
| clusteringA_tm | 0.3801 | 0.4611 | 0.064 | | -1.27 | 0.3587 | 0.4307 | 0.07 | -1.033 |
| clusteringA_cm | 0 | 0.0124 | 0.012 | | -1.02 | 0 | 0.0145 | 0.013 | -1.159 |
| clusteringA_ti | 0.1294 | 0.1368 | 0.023 | | -0.323 | 0.1344 | 0.1397 | 0.024 | -0.222 |
| clusteringA_to | 0.1504 | 0.1258 | 0.019 | | 1.328 | 0.1423 | 0.1182 | 0.017 | 1.388 |
|  | Mahalanobis distance = 5393 | | | | | Mahalanobis distance = 4493 | | | |

Table S7. Difference (percentage) in unnormalised ERGM parameter values from 1980s to 2000s. Exploitative competition is included only in one of the models (Offshore 1980s), difference can thus not be calculated.

| Configuration | Offshore | Coast |
| --- | --- | --- |
| Generalist | 19.12 | 12.33 |
| Highly predated species | -23.47 | -3.78 |
| Keystone species | -4.27 | 26.77 |
| Omnivory | -8.05 | -12.00 |
| Tri-trophic food chain | 12.11 | -19.45 |
| Exploitative competition | - |  |
| Apparative competition | -0.27 | -33.39 |

Table S8. Baltic Sea offshore 2000s alternative model (model specification shown in parameter estimates in the end of the table). This ERGM has the same model specification as the offshore 1980s food web. It can replicate the structural features better than the model specification used in the study, but this model is unstable in significant configurations (AT-U) and thus not a good model. Therefore, it was not selected for the study.

| Network configuration | Observed food web | | Random network, mean | | St. err. | | T-ratio |
| --- | --- | --- | --- | --- | --- | --- | --- |
| ArcA | 115 | | 109.44 | | 14.148 | | 0.393 |
| ReciprocityA | 0 | | 2.914 | | 1.712 | | -1.702 |
| In2StarA | 367 | | 296.675 | | 109.947 | | 0.64 |
| Out2StarA | 286 | | 253.346 | | 88.972 | | 0.367 |
| In3StarA | 984 | | 684.996 | | 461.259 | | 0.648 |
| Out3StarA | 630 | | 475.621 | | 295.627 | | 0.522 |
| TwoPathA | 273 | | 209.063 | | 37.045 | | 1.726 |
| Transitive-TriadA | 76 | | 65.802 | | 30.44 | | 0.335 |
| Cyclic-TriadA | 0 | | 1.278 | | 1.325 | | -0.965 |
| T1A | 0 | | 0 | | 0 | | NaN |
| T2A | 0 | | 0.096 | | 0.311 | | -0.309 |
| xT3A | 0 | | 0.697 | | 1.052 | | -0.662 |
| T4A | 0 | | 2.233 | | 2.508 | | -0.89 |
| T5A | 0 | | 1.397 | | 1.548 | | -0.903 |
| T6A | 0 | | 0.407 | | 0.761 | | -0.535 |
| T7A | 0 | | 18.548 | | 12.498 | | -1.484 |
| T8A | 0 | | 13.816 | | 10.093 | | -1.369 |
| SinkA | 3 | | 1.97 | | 1.078 | | 0.955 |
| SourceA | 3 | | 4.212 | | 1.605 | | -0.755 |
| IsolateA | 0 | | 0.261 | | 0.526 | | -0.496 |
| AinSA | 143.2139 | | 131.5946 | | 28.628 | | 0.406 |
| AoutSA | 135.0042 | | 124.5243 | | 26.929 | | 0.389 |
| AinSA2 | 143.2139 | | 131.5946 | | 28.628 | | 0.406 |
| AoutSA2 | 135.0042 | | 124.5243 | | 26.929 | | 0.389 |
| AinAoutSA | 61.213 | | 61.31 | | 5.318 | | -0.018 |
| ATA-T | 46.8589 | | 48.914 | | 16.812 | | -0.122 |
| ATA-C | 0 | | 3.7294 | | 3.804 | | -0.98 |
| ATA-D | 54.375 | | 48.3941 | | 15.635 | | 0.383 |
| ATA-U | 63.5 | | 54.6065 | | 21.293 | | 0.418 |
| A2PA-T | 171.7866 | | 170.81 | | 22.438 | | 0.044 |
| A2PA-D | 203.0332 | | 194.1215 | | 45.257 | | 0.197 |
| A2PA-U | 255.4375 | | 235.4543 | | 63.518 | | 0.315 |
| stddev_  indegreeA | 3.7523 | | 3.148 | | 0.65 | | 0.929 |
| skew_  indegreeA | 1.1831 | | 0.9617 | | 0.343 | | 0.646 |
| stddev_outdegreeA | 2.9461 | | 2.6756 | | 0.534 | | 0.507 |
| skew_out  degreeA | 1.4262 | | 0.8255 | | 0.325 | | 1.848 |
| clusteringA_tm | 0.2784 | | 0.3056 | | 0.096 | | -0.284 |
| clusteringA_cm | 0 | | 0.0191 | | 0.02 | | -0.947 |
| clusteringA_ti | 0.1035 | | 0.1087 | | 0.017 | | -0.302 |
| clusteringA_to | 0.1329 | | 0.1271 | | 0.023 | | 0.258 |
|  | Mahalanobis distance = 3473 | | | | | | |
| **PARAMETER ESTIMATES** | | | | | | | |
| Configuration | | Parameter estimate | | St Err | | Significance | |
| ArcA | | -5.7142 | | 2.07 | | * | |
| AinSA | | 1.931 | | 0.644 | | * | |
| AoutSA | | 1.2629 | | 0.657 | |  | |
| AinAoutSA | | 2.4891 | | 0.762 | | * | |
| ATA-U | | 0.3116 | | 0.142 | | * | |
| A2PA-T | | -0.6698 | | 0.086 | | * | |
| A2PA-D | | -0.1495 | | 0.054 | | * | |
| A2PA-U | | -0.1423 | | 0.051 | | * | |

#

Table S9. Results for the Baltic Sea descriptive motif approach analyses

| **Motif** | **real** | **rand** | **srand** | **z-score** |
| --- | --- | --- | --- | --- |
| **OFFSHORE 1980s** | | | | |
| D1 | 2 | 0.581 | 0.656 | 2.164 |
| D2 | 4 | 1.724 | 1.009 | 2.255 |
| D3 | 4 | 5.962 | 1.481 | -1.324 |
| D4 | 6 | 9.676 | 2.052 | -1.792 |
| D5 | 0 | 0.787 | 0.873 | -0.901 |
| D6 | 0 | 0 | 0 |  |
| D7 | 0 | 0.089 | 0.285 | -0.312 |
| D8 | 1 | 0.911 | 0.285 | 0.312 |
| S1 | 144 | 120.039 | 6.001 | 3.993 |
| S2 | 59 | 75.535 | 5.738 | -2.882 |
| S3 | 0 | 2.213 | 1.509 | -1.466 |
| S4 | 179 | 163.884 | 5.782 | 2.614 |
| S5 | 254 | 239.741 | 5.764 | 2.474 |
| **OFFSHORE 2000s** | | | | |
| D1 | 0 | 0 | 0 |  |
| D2 | 0 | 0 | 0 |  |
| D3 | 0 | 0 | 0 |  |
| D4 | 0 | 0 | 0 |  |
| D5 | 0 | 0 | 0 |  |
| D6 | 0 | 0 | 0 |  |
| D7 | 0 | 0 | 0 |  |
| D8 | 0 | 0 | 0 |  |
| S1 | 197 | 175.626 | 7.085 | 3.017 |
| S2 | 76 | 85.995 | 6.334 | -1.578 |
| S3 | 0 | 3.793 | 1.971 | -1.925 |
| S4 | 210 | 200.005 | 6.334 | 1.578 |
| S5 | 291 | 281.005 | 6.334 | 1.578 |
| **COAST 1980s** | | | | |
| D1 | 0 | 1.103 | 0.689 | -1.601 |
| D2 | 1 | 1.331 | 0.801 | -0.413 |
| D3 | 12 | 8.42 | 1.531 | 2.338 |
| D4 | 8 | 5.964 | 1.674 | 1.216 |
| D5 | 0 | 1.374 | 0.961 | -1.43 |
| D6 | 0 | 0 | 0 |  |
| D7 | 0 | 0 | 0 |  |
| D8 | 0 | 0 | 0 |  |
| S1 | 351 | 280.191 | 9.221 | 7.679 |
| S2 | 217 | 268.576 | 9.644 | -5.348 |
| S3 | 0 | 5.953 | 3.016 | -1.974 |
| S4 | 503 | 450.321 | 9.667 | 5.449 |
| S5 | 618 | 566.093 | 9.645 | 5.382 |
| **COAST 2000s** | | | | |
| D1 | 0 | 0 | 0 |  |
| D2 | 0 | 0 | 0 |  |
| D3 | 0 | 0 | 0 |  |
| D4 | 0 | 0 | 0 |  |
| D5 | 0 | 0 | 0 |  |
| D6 | 0 | 0 | 0 |  |
| D7 | 0 | 0 | 0 |  |
| D8 | 0 | 0 | 0 |  |
| S1 | 404 | 330.611 | 10.237 | 7.169 |
| S2 | 226 | 275.092 | 9.904 | -4.957 |
| S3 | 0 | 8.099 | 3.408 | -2.376 |
| S4 | 568 | 518.908 | 9.904 | 4.957 |
| S5 | 615 | 565.908 | 9.904 | 4.957 |
| Number of randomization = 1000 | | | | |

# 4. Figures

#


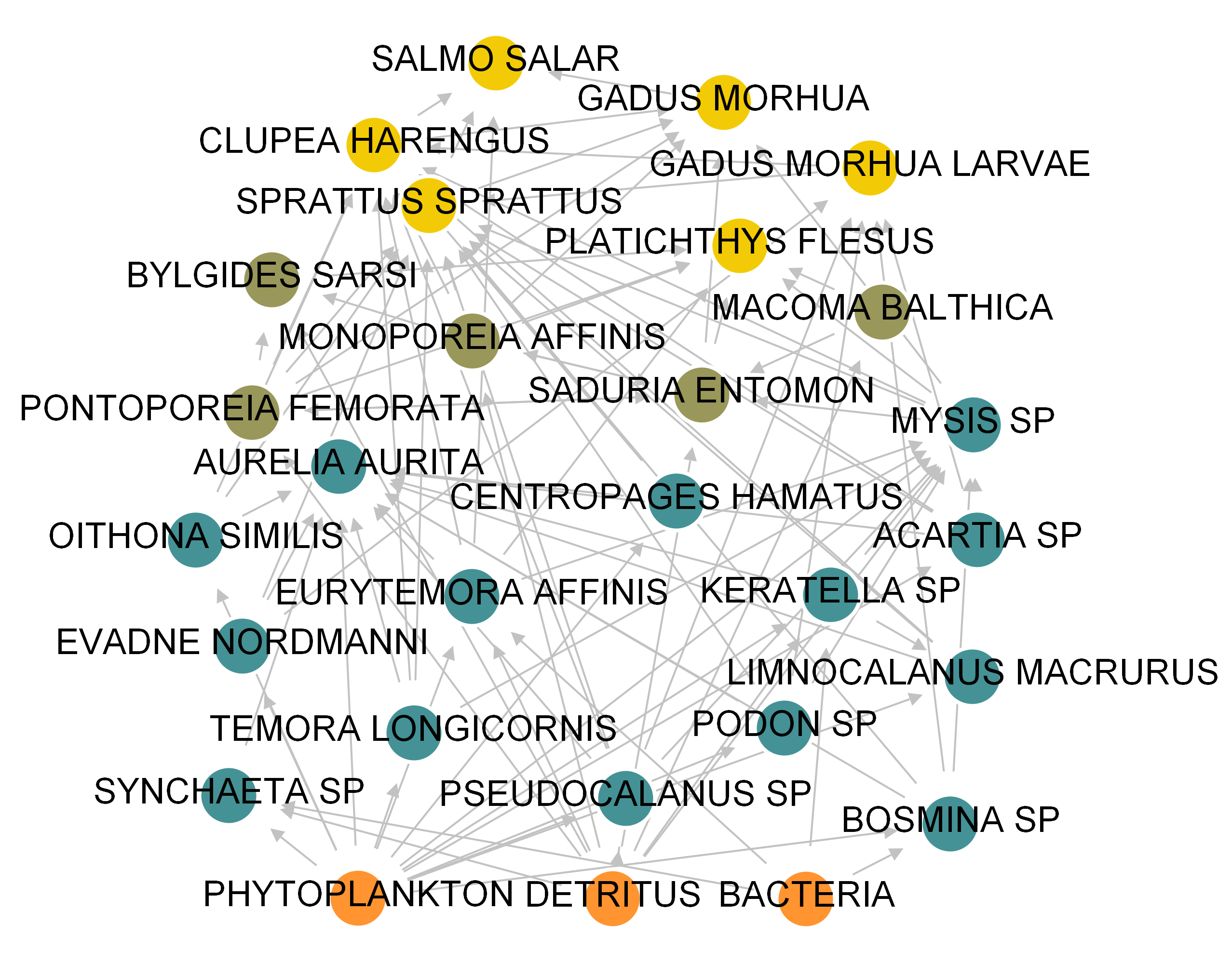


a.


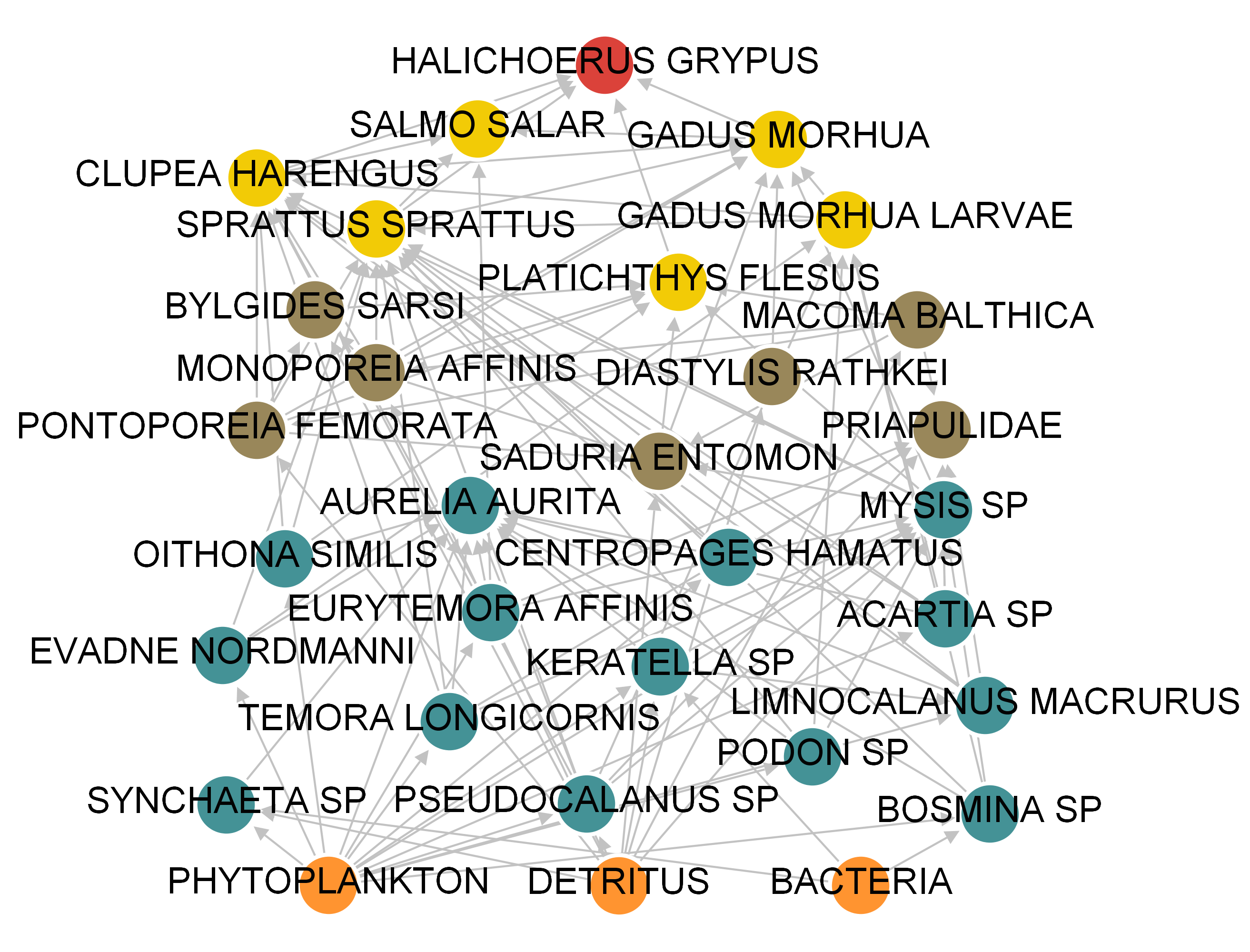


b.


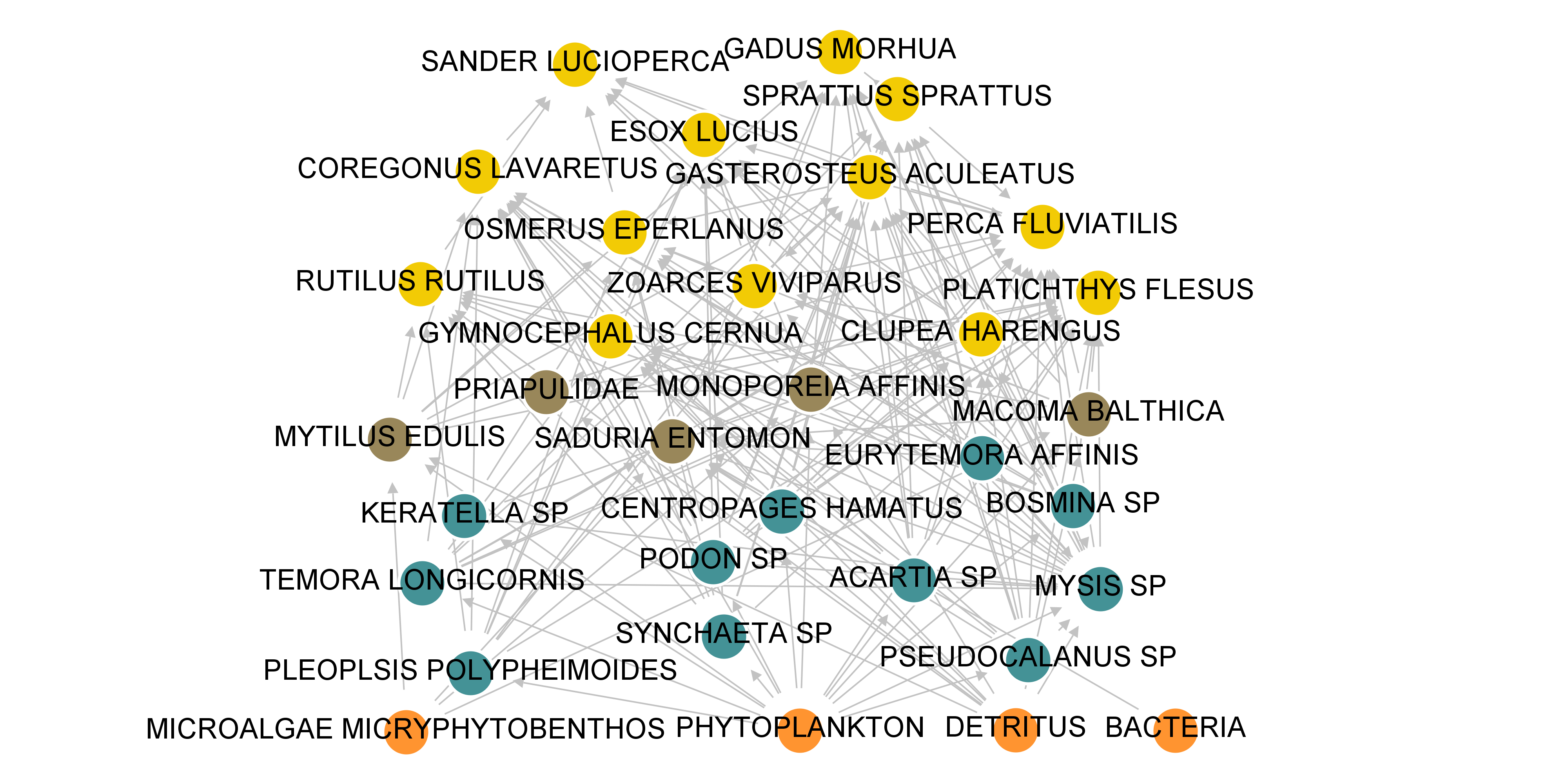


c.


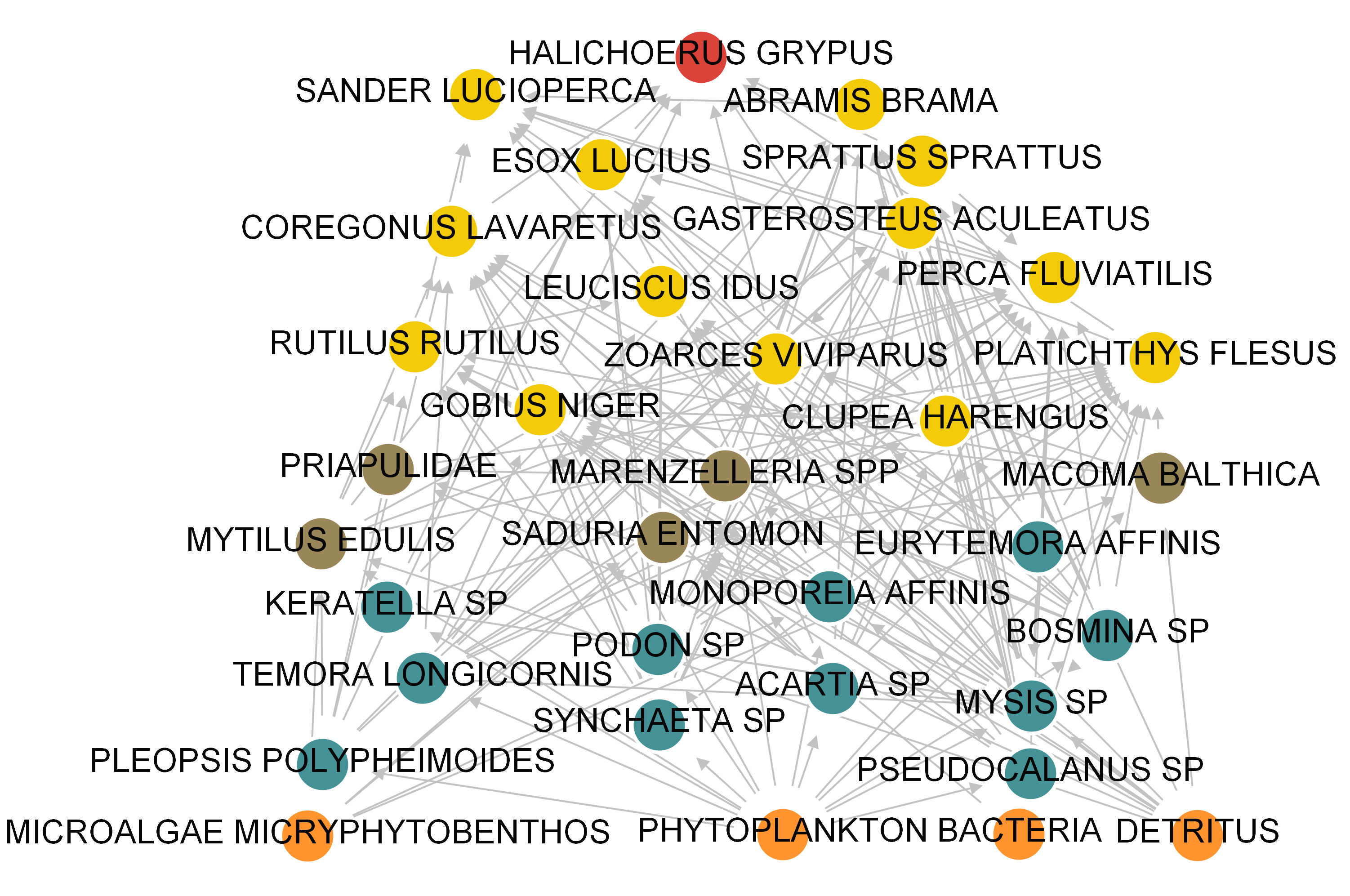


d.

Figure S1a-d. Baltic Sea food webs used in the study. a) Offshore 1980s; b) Offshore 2000s; c) Coast 1980s; d) Coast 2000s.


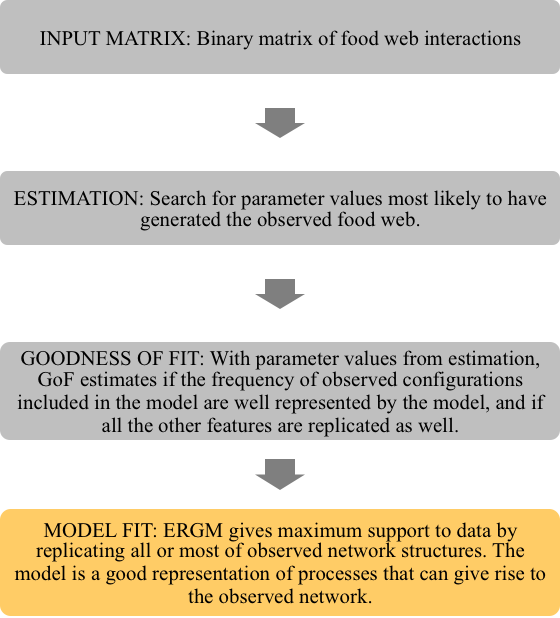


No convergence: ERGM cannot find solution with chosen specification.

Extreme GoF T-ratio values: chosen parameters cannot explain the observed network.

Model specification

Model convergence

Figure S2. ERGM workflow. ERGM is a method to examine network empirically. Researcher specifies the model based on relevant theory or hypothesis. If the model does not convergence the specification must be modified. In the case of model convergence, GoF procedure evaluates the model fit for all ERGM configurations. If extreme t-ratios occur, the values of GoF can be examined to get insights for improving the model specification to gain model fit.

REFERENCES

1. Lusher, D., Koskinen, J. & Robins, G., editors 2013 *Exponential Random Graph Models for Social Networks. Theory, Methods and Applications.* Cambridge, UK: Cambridge University Press.

2. FIRM Finnish Institute of Marine Research 2007 *FIMR Monitoring of the Baltic Sea Environment - Annual Report 2006. MERI. Report Series of the Finnish Institute of Marine Research No 59, 2007.* Helsinki: Finnish Institute of Marine Research.

3. Möllmann, C., Diekmann, R., Müller-Karulis, B., Kornilovs, G., Plikshs, M. & Axe, P. 2009 Reorganization of a large marine ecosystem due to atmospheric and anthropogenic pressure: a discontinuous regime shift in the Central Baltic Sea. *Glob. Chang. Biol.* **15**, 1377–1393. (doi:10.1111/j.1365-2486.2008.01814.x)

4. Harding, K. C., Härkönen, T., Helander, B. & Karlsson, O. 2013 Status of Baltic grey seals: Population assessment and extinction risk. *NAMMCO Sci. Publ.* **6**, 33–56. (doi:10.7557/3.2720)

5. Hiby, L., Lundberg, T., Karlsson, O., Watkins, J. & Jüssi, M. 2005 Estimates of the size of the Baltic grey seal population based on photo-identification data. *NAMMCO Sci. Publ.* **6**, 163–175.

6. Möllmann, C., Conversi, A. & Edwards, M. 2011 Comparative analysis of European wide marine ecosystem shifts: a large-scale approach for developing the basis for ecosystem-based management. *Biol. Lett.* **7**, 484–6. (doi:10.1098/rsbl.2010.1213)

7. Österblom, H., Hansson, S., Larsson, U., Hjerne, O., Wulff, F., Elmgren, R. & Folke, C. 2007 Human-induced Trophic Cascades and Ecological Regime Shifts in the Baltic Sea. *Ecosystems* **10**, 877–889. (doi:10.1007/s10021-007-9069-0)

8. Casini, M., Lövgren, J., Hjelm, J., Cardinale, M., Molinero, J.-C. & Kornilovs, G. 2008 Multi-level trophic cascades in a heavily exploited open marine ecosystem. *Proc. Natl. Acad. Sci. U. S. A.* **275**, 1793–801. (doi:10.1098/rspb.2007.1752)

9. Möllmann, C. & Köster, F. W. 1999 Food consumption by clupeids in the Central Baltic: evidence for top-down control? *ICES J. Mar. Sci.* **56**, 100–113. (doi:10.1006/jmsc.1999.0630)

10. Köster, F. W. & Möllmann, C. 2000 Trophodynamic control by clupeid predators on recruitment success in Baltic cod? *ICES J. Mar. Sci.* **57**, 310–323. (doi:10.1006/jmsc.1999.0528)

11. Nyström, M. et al. 2012 Confronting feedbacks of degraded marine ecosystems. *Ecosystems* **15**, 695–710. (doi:10.1007/s10021-012-9530-6)

12. Alheit, J. 2007 Consequences of regime shifts for marine food webs. *Int. J. Earth Sci.* **98**, 261–268. (doi:10.1007/s00531-007-0232-9)

13. Tomczak, M. T., Niiranen, S., Hjerne, O. & Blenckner, T. 2012 Ecosystem flow dynamics in the Baltic Proper—Using a multi-trophic dataset as a basis for food–web modelling. *Ecol. Modell.* **230**, 123–147. (doi:10.1016/j.ecolmodel.2011.12.014)

14. Rousi, H. et al, Laine, a. O., Peltonen, H., Kangas, P., Andersin, A.-B., Rissanen, J., Sandberg-Kilpi, E. & Bonsdorff, E. 2013 Long-term changes in coastal zoobenthos in the northern Baltic Sea: the role of abiotic environmental factors. *ICES J. Mar. Sci.* (doi:10.1093/icesjms/fss197)

15. Weigel, B., Andersson, H. C., Meier, H. E. M., Blenckner, T., Snickars, M. & Bonsdorff, E. 2015 Long-term progression patterns and drivers of coastal zoobenthos in a changing system. *Mar. Ecol. Prog. Ser.* **528**, 141–159.

16. Snickars, M., Weigel, B. & Bonsdorff, E. 2014 Impact of eutrophication and climate change on fish and zoobenthos in coastal waters of the Baltic Sea. *Submitt. Mar. Biol.* **162**, 141–151. (doi:10.1007/s00227-014-2579-3)

17. Reid, P. C. et al. 2015 Global impacts of the 1980s regime shift. *Glob. Chang. Biol.* , n/a–n/a. (doi:10.1111/gcb.13106)

18. Camacho, J., Stouffer, D. & Amaral, L. 2007 Quantitative analysis of the local structure of food webs. *J. Theor. Biol.* **246**, 260–268. (doi:10.1016/j.jtbi.2006.12.036)

19. Stouffer, D. B., Camacho, J., Guimera, R., Ng, C. A. & Nunes Amaral, L. A. 2005 Quantitative patterns in the structure of model and empirical food webs. *Ecology* **86**, 1301–1311.

20. Snijders, T. & Pattison, P. 2006 New specifications for exponential random graph models. *Sociol. Methodol.* , 99–153.

21. Milo, R., Shen-Orr, S., Itzkovitz, S., Kashtan, N., Chklovskii, D. & Alon, U. 2002 Network motifs: simple building blocks of complex networks. *Science* **298**, 824–7. (doi:10.1126/science.298.5594.824)

22. Stouffer, D. B., Camacho, J., Jiang, W., Amaral, L. a N. & Nunes Amaral, L. A. 2007 Evidence for the existence of a robust pattern of prey selection in food webs. *Proc. R. Soc. B* **274**, 1931–1940. (doi:10.1098/rspb.2007.0571)

23. Wang, P., Robins, G. & Pattison, P. 2009 *PNet. Program for the Simulation and Estimation of Exponential Random Graph (p*) Models. User manual.* Australia.: Department of Psychology. School of Behavrioral Science. University of Melbourne.

24. Pimm, S. L., Lawton, J. H. & Cohen, J. E. 1991 Food web patterns and their consequences. *Nature* **350**, 669–674. (doi:10.1038/350669a0)
